# Factors important for health-related quality of life in men and women: The population based SCAPIS study

**DOI:** 10.1371/journal.pone.0294030

**Published:** 2023-11-03

**Authors:** Max Olsson, Anders J. Björkelund, Jacob Sandberg, Anders Blomberg, Mats Börjesson, David Currow, Andrei Malinovschi, Magnus Sköld, Per Wollmer, Kjell Torén, Carl-Johan Östgren, Gunnar Engström, Magnus Ekström

**Affiliations:** 1 Lund University, Faculty of Medicine, Department of Clinical Sciences Lund, Respiratory Medicine, Allergology and Palliative Medicine, Lund, Sweden; 2 Lund University, Faculty of Science, Centre for Environmental and Climate Science (CEC), Lund, Sweden; 3 Umeå University, Department of Public Health and Clinical Medicine, Umeå, Sweden; 4 Dept of Molecular and Cardiovascular Medicine, Center for Health and Performance, Sahlgrenska Academy; 5 Dept MGAÖ, Sahlgrenska University Hospital, Gothenburg, Sweden; 6 Faculty of Science, Medicine and Health, University of Wollongong, Wollongong, New South Wales, Australia; 7 Dept of Medical Sciences, Clinical Physiology, Uppsala University, Uppsala, Sweden; 8 Karolinska Institutet, Department of Medicine Solna, Stockholm, Sweden; 9 Department of Respiratory Medicine and Allergy, Karolinska University Hospital, Stockholm, Sweden; 10 Department of Translational Medicine, Lund University, Malmö, Sweden; 11 Occupational and Environmental Medicine, School of Public Health and Community Medicine, Institute of Medicine, Sahlgrenska Academy, University of Gothenburg, Gothenburg, Sweden; 12 Department of Health, Medicine and Caring Sciences, Linköping University, Linkoping, Sweden; 13 Lund University, Faculty of Medicine, Department of Clinical Sciences Malmö, Cardiovascular Epidemiology, Malmö, Sweden; Hamasaki Clinic, JAPAN

## Abstract

**Introduction:**

Health-related quality of life (HRQoL) is essential for human wellbeing, influenced by a complex interplay of factors, and is reported lower in women than men. We aimed to evaluate which factors were the most important for HRQoL in a middle-aged general population.

**Methods:**

This was a cross-sectional, multi-centre study of 29,212 men (48%) and women (52%) aged 50–64 in the general population in Sweden. Physical and mental HRQoL (0–100) was assessed using the Short Form 12 questionnaire, and association was evaluated for 356 variables including demographics, lifestyle, symptoms, physiological measurements, and health conditions. Using machine learning, each variable´s importance for HRQoL was measured by an importance score, comparable to effect size, and summarised in 54 factors, in men and women separately.

**Results:**

Men and women had similar mean and standard deviation (SD) scores for physical HRQoL (53.4 [SD 8.1] vs 51.4 [9.7]) and mental HRQoL (37.1 [5.0] vs 37.3 [5.4]). The most important factors for physical HRQoL were (importance score) physical activity (40), employment (36), pain (33), sleep (33), and sense of control (26). The most important factors for mental HRQoL were sense of control (18), physical activity (12), depression (12), pain (6), and employment (5).

**Conclusions:**

The factors important for HRQoL identified by this study are likely to be amenable to interventions, and our findings can support prioritising interventions. The identified factors need to be a target even before middle-age to lay the foundation for long and happy lives.

## Introduction

Health comprises multiple dimensions and is defined by the World Health Organisation (WHO) as “*a state of complete physical*, *mental and social well-being and not merely the absence of disease or infirmity*” [1]. Health-related quality of life (HRQoL) is an essential aspect of human life and a key outcome in public health research, interventions and policy [2]. HRQoL is usually self-reported through questionnaires, and different aspects of HRQoL are often derived to represent the WHO’s definition of health’s multiple dimensions [3]. HRQoL is a good measure of overall health as it captures information of both physical and mental health [4], which could be more important outcomes for the individuals compared to health conditions and physiological measurements of the body. HRQoL is also an important predictor of survival [5], which means that HRQoL is important to measure and monitor. Decreased HRQoL is described as the result from suffering from health conditions and reflects individuals’ adaptation to the adverse health outcomes of health conditions [6], wherefore understanding which factors that affect HrQoL is important for public health decisions. HRQoL is likely determined by the interplay of multiple physical, mental and socioeconomic factors, and this complex interplay must be accounted for in analyses to fully understand differences in HRQoL [7, 8]. In the middle age, the prevalence of diseases and troublesome symptoms starts to increase [6], which can significantly decrease HRQoL, and the factors explaining variance in HRQoL can be studied meaningfully. It is important to identify factors that can be modified in younger and middle-aged populations to be able to improve future health by public health policies and interventions during and, especially, before reaching an older age.

Differences in HRQoL have been reported between the sexes, with women often reporting poorer HRQoL [8, 9]. However, knowledge is limited on which factors are most strongly related to HRQoL and whether they differ between men and women [10]. A limitation of previous population studies is that associations with HRQoL were mainly studied for one or a few factors without comparing the relative importance of each factor. There is a need for studies using methods that consider the complexity of multiple factors associated with HRQoL and studies that account for potential nonlinear associations.

Machine learning is a data-driven subtype of artificial intelligence (AI) that can analyse large datasets and identify complex nonlinear associations between factors and outcomes beyond the capabilities of traditional statistical models [11]. Identifying important factors for HRQoL and comparing them between men and women is an important method for reducing any HRQoL gaps between the sexes and identifying public health target areas. Also, a hypothesis-free machine learning approach could identify factors related to differences in HRQoL which could be compared to previous findings and generate new hypothesis for future studies.

The primary aim of this study was to evaluate the most important factors related to physical and mental HRQoL among middle-aged men and women in the general population using machine learning. The secondary aim was to compare the shapes of the associations between identified important factors and HRQoL.

## Materials and methods

### Study design and population

This was a national, population-based, cross-sectional study of individuals aged 50 to 64 years in the Swedish CArdioPulmonary bioImage Study (SCAPIS) [12]. Between 2013 and 2018, the SCAPIS study collected data from six different sites in Sweden (Uppsala, Umeå, Linköping, Malmö/Lund, Gothenburg and Stockholm), as previously described [12]. The SCAPIS study aimed to include a national representative sample between age groups and the sexes. The study randomly recruited participants from the general population stratified by age (50–54, 55–59 and 60–64 years) and sex. The participants were asked to complete an extensive questionnaire about health-related factors and a number of assessments and tests at the study sites. Participants who were unable to understand spoken and written Swedish could not provide consent to participate in the study and were thus not included in the SCAPIS study. The exclusion criteria for the present analysis were missing data on the Short Form 12 (SF-12) [3] regarding physical and mental HRQoL. A validation of the generalisability of the SCAPIS cohort indicates that, with the exception of increased alcohol consumption, the participants are representative of the Swedish general population [13]. The data from the SCAPIS database were accessed and analysed in the present study between February and May 2022.

### Assessments

HRQoL was measured using the validated Swedish version of the SF-12, which comprises 12 items measuring eight different aspects of HRQoL that are calculated into physical and mental HRQoL component scores. The SF-12 for physical and mental scores range from 0 to 100, with higher scores reflecting better HRQoL [14].

Evaluated factors comprised 356 variables spanning demographics, socioeconomics, lifestyle, anthropometrics, symptoms, physiological measurements, blood tests, treatments, and health conditions (see S1 Table for a full list of variables). The following variables were self-reported: employment variables (extent of gainful employment, work situation), socioeconomic variables (ability to find money for unforeseen events, housing type), sleep variable (length, quality, chance of dozing during the day, snoring), physical activity (extent at work, [15] during leisure time [16], and mode of travel to work), breathlessness (Medical research council [MRC] breathlessness scale 1–5 [17]), sense of control (extent of agreement of seven statements), [18] and depression (eight yes/no questions of common depression symptoms) [18]. Pain was measured as the usage of nonprescription pain-reliving drugs (number of doses of nonprescription medication weekly). Socioeconomic information was also collected based on the participants’ neighbourhood data (ratio of foreign individuals, income, education, employment ratio); these data were taken from Statistics Sweden. Physical activity was also assessed by accelerometry for one week and comprised the percentage of wear time, total minutes, and average minutes of low, moderate, moderate to vigorous, sedentary, and vigorous intensity physical activity [19]. Body size was measured as height, weight, body mass index (BMI), hip/waist circumference and waist-hip ratio. Because of the large number of variables in the present study, the variables were categorised into 54 different factors to present similar domains related to HRQoL. The categorisation was decided in discussion among the authors before the analysis was conducted, meaning that the categorisation did not affect the data analysis and was used only for simplifying the interpretation. S1 Table shows a full list of the categorisation.

### Machine learning

Supervised machine learning [11] was used to predict physical and mental HRQoL among the participants. To evaluate a model’s ability to predict an outcome, we split the study population into a *training* set and a *test* set. During the *training phase*, the model learned to predict the outcome by learning to identify patterns in the training set. The model was improved by tuning hyperparameters, which can be conceived as different settings of a model, for example learning rate and tree depth. These hyperparameters can be adjusted to improve prediction performance. The models’ hyperparameters were evaluated by tenfold cross-validation on the training sets before deciding the sets of hyperparameters to be used in the final models. The final model’s ability to predict HRQoL using unseen data (external validity) was evaluated by measuring performance to predict the outcome on the test set. The test set was kept isolated from the model during the whole training phase to evaluate the generalisability of the study results. A similar machine learning methodology was used in a previous pilot study exploring important factors for self-perceived health in older mens [20]. S1 Fig shows an overview of the data processing in the present study. Before the analysis, data from five SCAPIS study sites (Uppsala, Umeå, Linköping, Malmö/Lund, Gothenburg) were pooled and assigned as the training set (n = 24,247). Data from the remaining study site, Stockholm (n = 4,965; 17%), were used as the independent test set [11].

### Statistical analyses

Baseline patient characteristics were summarised using basic descriptions. The machine learning algorithm extreme gradient-boosting (XGBoost) [21] was used to predict physical and mental component scores of HRQoL. XGBoost has been shown to perform better than other algorithms on tabular data [21]. All factors (except the outcomes) were used in four separate XGBoost models to predict physical and mental HRQoL for men and women separately. No missing values of the factors were imputed, as the XGBoost algorithm is able to handle missing values directly [21].

The models’ hyperparameters were evaluated by tenfold cross-validation on the training sets. Hyperparameters with the best performance from the cross-validations were reported and used to train the final models on the training sets. The final models’ ability to generalise for unseen data was evaluated by their mean absolute errors (MAE) when predicting the HRQoL outcomes among men and women on the test sets. A lower MAE corresponds to better generalisability of the model. When evaluated on the test set, the physical HRQoL model for men had an MAE of 4.09, and the physical HRQoL model for women had an MAE of 4.95. The mental HRQoL model for men had an MAE of 3.50, and the mental HRQoL model for women had an MAE of 3.99 when evaluated on the test set. Taken together, these findings indicate that all models were generalisable for unseen data and that the importance of factors could be generalised for the whole study sample. The final model hyperparameters are shown in S2 Table.

### Importance of factors for HRQoL

Associations between variables and physical and mental components of HRQoL were analysed using Shapley Additive exPlanations (SHAP) scores [22]. SHAP has emerged as a robust and unbiased method to assess the strength of associations in studies using machine learning models [22]. SHAP scores are adjusted for all other variables in the analysis. SHAP scores correspond to the degree of change from the mean of an outcome variable by a predictor variable in individual participants [22]. The same variable can have different SHAP scores, both positive or negative, for different participants depending on the participants’ other variables. For example, depending on an individual’s characteristics, such as age and sex, a participant’s BMI value can have different associations (and thus, SHAP scores) with worse or better HRQoL in the individual.

To quantify the importance of variables for participants’ HRQoL, importance scores were calculated for each variable. The importance score was calculated as the absolute mean of the participants’ SHAP for each variable divided by the MAE of each model, which is comparable to effect size. The importance scores of the variables were then summarised into 54 factors. To rank the most important factors for physical and mental HRQoL for all participants, the mean of the factors’ importance scores was calculated among men and women. Ninety-five percent confidence intervals (CI) were first calculated for all participants and then separately for men and women using one sample t-tests.

### Shapes of associations with HRQoL

The strength of the association between each factor and physical and mental HRQoL was explored using scatterplots with SHAP scores as the Y-axis and the values of the variables as the X-axis. To simplify the interpretation of the scatterplots, two locally estimated scatterplot smoothing lines (LOESS) were added to the scatterplots for each sex.

The analyses were conducted using the software R version 4.1.2 (R Foundation for Statistical Computing, Austria) with the packages XGBoost version 1.5.0.2 and SHAPforxgboost version 0.1.1.

### Ethical considerations

The SCAPIS study was approved as a multicentre study by the ethics committee at Umeå University (Dnr 2010–228–31 M). The present analysis was approved by the Swedish Ethical Review Authority (Dnr 2021–00288). All participants provided their written informed consent.

## Results

### Participant characteristics

After excluding 522 men and 420 women due to missing data on physical or mental HRQoL, a total of 14,124 (48%) men and 15,088 (52%) women were included in the analyses. Participant characteristics are shown in Table 1. The mean (SD) age was 57.5 (4.3) years, the mean physical HRQoL score was 52.4 (9.0) and the mean mental HRQoL score was 37.2 (5.2). Approximately one-half (48%) reported sleeping well or very well, and one-third (28%) reported experiencing sadness/depression, almost one-half (44%) reported being regular or former smokers, and almost one-half (42%) reported exercising moderately/heavily regularly. The majority of the participants (73%) were married, born in Sweden (84%), and employed (87%). The most common level of education was secondary school (45%) or university (45%). Men and women had similar mean HRQoL scores for both physical HRQoL (53.4 [SD 8.1] vs. 51.4 [9.7]) and mental HRQoL (37.1 [5.0] vs. 37.3 [5.4]).

**Table 1:** Characteristics of 29,212 men and women aged 50–64 in the general population.

Variable (% missing observations)	All	Women	Men
**N**	29,212	15,088 (52%)	14,124 (48%)
**SF-12 physical HRQoL (0%)**	52.4 (9.0)	51.4 (9.7)	53.4 (8.1)
**SF-12 mental HRQoL (0%)**	37.2 (5.2)	37.3 (5.4)	37.1 (5.0)
Age (0%)	57.5 (4.4)	57.5 (4.3)	57.5 (4.4)
Married (1%)	21389 (73%)	10457 69%)	10932 (77%)
Born in Sweden (0%)	24463 (84)	12576 (83%)	11887 (84%)
**Education** (0%)			
No elementary school	189 (1%)	92 (1%)	97 (1%)
Elementary school	2519 (9%)	1134 (8%)	1385 (10%)
Upper secondary school	13233 (45%)	6390 (42%)	6843 (48%)
University degree	13161 (45%)	7421 (49%)	5740 (41%)
**Employment professional work (1%)**	25323 (87%)	12948 (86%)	12375 (88%)
**Difficulties managing regular expenses last 12 months (1%)**	1587 (5%)	864 (6%)	723 (5%)
**BMI (0%)**	27.0 (4.5)	26.5 (4.8)	27.4 (4.0)
**FEV1/FVC post bronchodilatation (1%)**	0.78 (0.1)	0.78 (0.1)	0.78 (0.1)
**Diastolic blood pressure (0%)**	77.5 (10.5)	76.6 (10.8)	78.5 (10.1)
**Pack-years of smoking (1%)**	7.7 (12.1)	7.6 (11.4)	7.7 (12.8)
**Frequency having an alcoholic drink, last 12 months (1%)**			
Never	2628 (9%)	1573 (10%)	1055 (7%)
Monthly or less	4514 (16%)	2689 (18%)	1825 (13%)
2 to 4 times a month	11094 (38%)	5719 (38%)	5375 (38%)
2 to 3 times a week	8733 (30%)	4282 (28%)	4451 (32%)
4 or more times a week	2218 (8%)	761 (5%)	1348 (10%)
**Current smoking status, self-reported (1%)**			
Regular	2218 (8%)	1224 (8%)	994 (7%)
Occasional	1418 (5%)	663 (4%)	755 (5%)
Former	10546 (36%)	5818 (39%)	4728 (33%)
Never	14650 (50%)	7178 (48%)	7472 (53%)
**Exertion (1%)**			
Sedentary	3422 (12%)	1610 (11%)	1812 (13%)
Moderate occasionally exercise	13376 (46%)	7350 (49%)	6026 (43%)
Moderate but regular exercise	8373 (29%)	4451 (30%)	3922 (28%)
Frequent heavy exercise	3886 (13%)	1598 (11%)	2282 (16%)
**Quality of sleep (1%)**			
Very well	5030 (17%)	2189 (15%)	2841 (20%)
Well	8919 (31%)	4114 (27%)	4805 (34%)
Rather well	10083 (35%)	5611 (37%)	4472 (32%)
Badly	4160 (14%)	2540 (17%)	1620 (11%)
Very badly	776 (3%)	517 (3%)	259 (2%)
**Self-reported health conditions**			
Allergic rhinitis (2%)	6348 (22%)	3335 (22%)	3013 (22%)
Asthma (1%)	2409 (8%)	1452 (10%)	957 (7%)
Cancer (1%)	1733 (6%)	1076 (7%)	657 (5%)
Chronic sinusitis (2%)	679 (2.3)	404 (3%)	275 (2%)
COPD, chronic bronchitis, or emphysema (1%)	272 (1%)	165 (1%)	107 (1%)
Feelings of sadness/depression, last 12 months (2%)	8069 (28%)	5204 (34%)	2865 (20%)
Diabetes (1%)	1266 (4%)	469 (3%)	797 (6%)
Heart failure (1%)	149 (1%)	52 (1%)	97 (1%)
Hypertension (1%)	6570 (23%)	3134 (21%)	3436 (24%)
Myocardial infarction (1%)	470 (2%)	110 (1%)	360 (3%)
Rheumatic disease (1%)	1073 (4%)	709 (5%)	364 (3%)
Stroke, doctor-diagnosed (1%)	417 (1%)	184 (1%)	233 (2%)
Urticaria (3%)	7469 (26%)	5153 (34%)	2316 (16%)

Data is presented as mean (SD) or n (%). Abbreviations: BMI = Body mass index; COPD = Chronic obstructive pulmonary disease; FEV1 = Forced Expiratory Volume in 1 second; FVC = forced vital capacity.

### Factor importance for physical HRQoL

**Fig 1:**
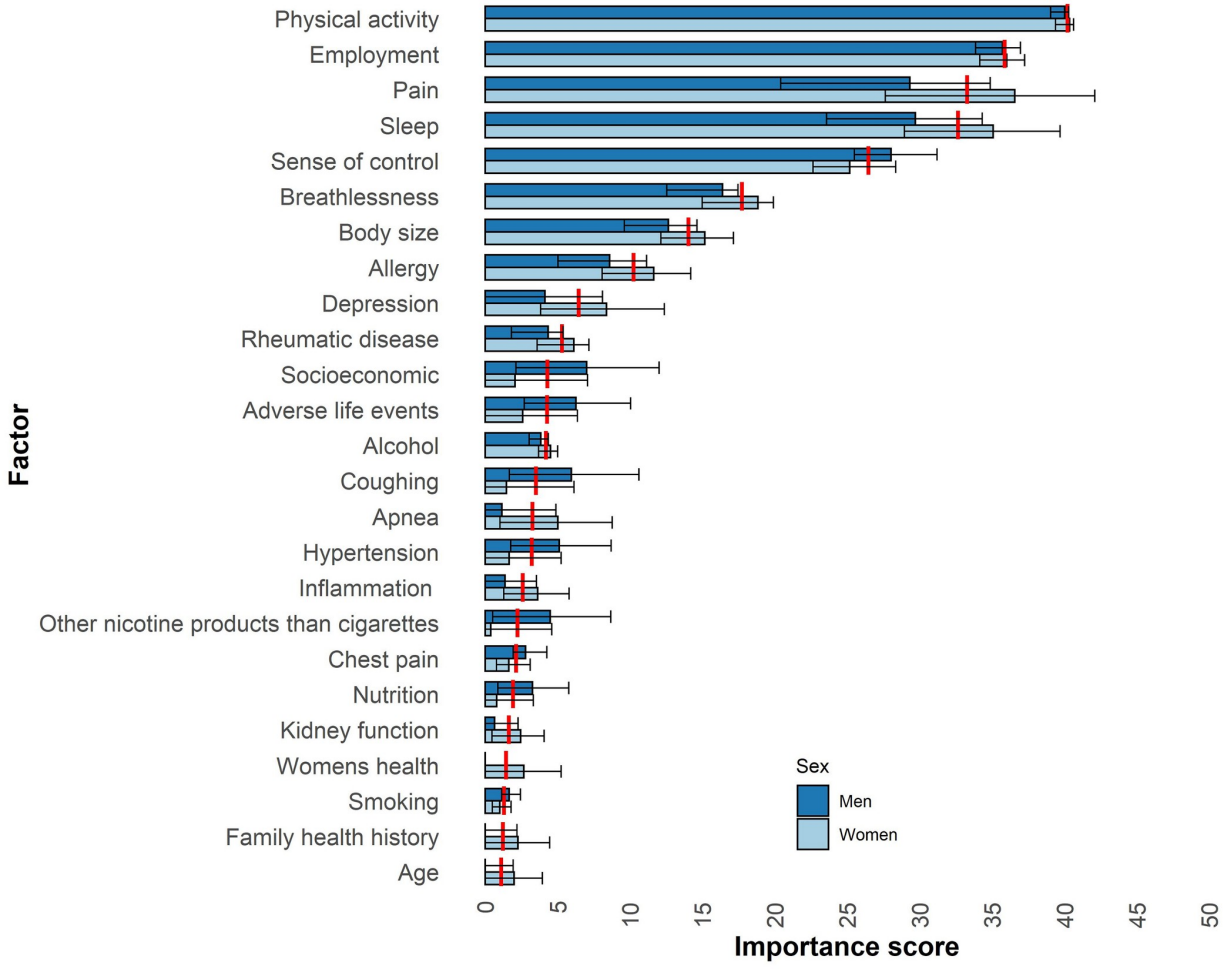
Factors related to physical HRQoL among men (n = 14,124) and women (n = 15,088) aged 50–64 in Sweden. The factors are the summarised importance scores of similar variables, which were assigned before the analysis. The importance score corresponds to the SHapley Additive exPlanations (SHAP) absolute mean divided by the models’ mean absolute error, which is comparable to the effect size. The SHAP absolute mean corresponds to the average degree of change from the mean score of the Short Form 12 for physical health-related quality of life (HRQoL; men: 53.4; women: 51.4) by a predictor variable among all participants. The plot shows the top 25 domains with highest importance scores for physical HRQoL among all participants and are ordered in descending order. The red line marks the average importance score for all participants. The error bars present the 95% confidence intervals calculated by t-tests.

The factors most important for physical HRQoL among men, women, and all participants are shown in Fig 1 as well as S3 Table. The most important factors for physical HRQoL for all participants (men and women analysed together) were as follows: physical activity (with an importance score of 40), employment (36), pain (33), sleep (33), sense of control (26), breathlessness (18), body size (14), and allergy (10). The order of the factors’ importance for physical HRQoL among the sexes was the same as for all participants, except for pain, which was ranked second among women and fourth among men (Fig 1).

There were some numerical differences between the sexes in the importance scores of the important factors for physical HRQoL. However, none of these differences were statistically significant, as indicated by the overlapping CIs shown in Fig 1.

### Factor importance for mental HRQoL

**Fig 2:**
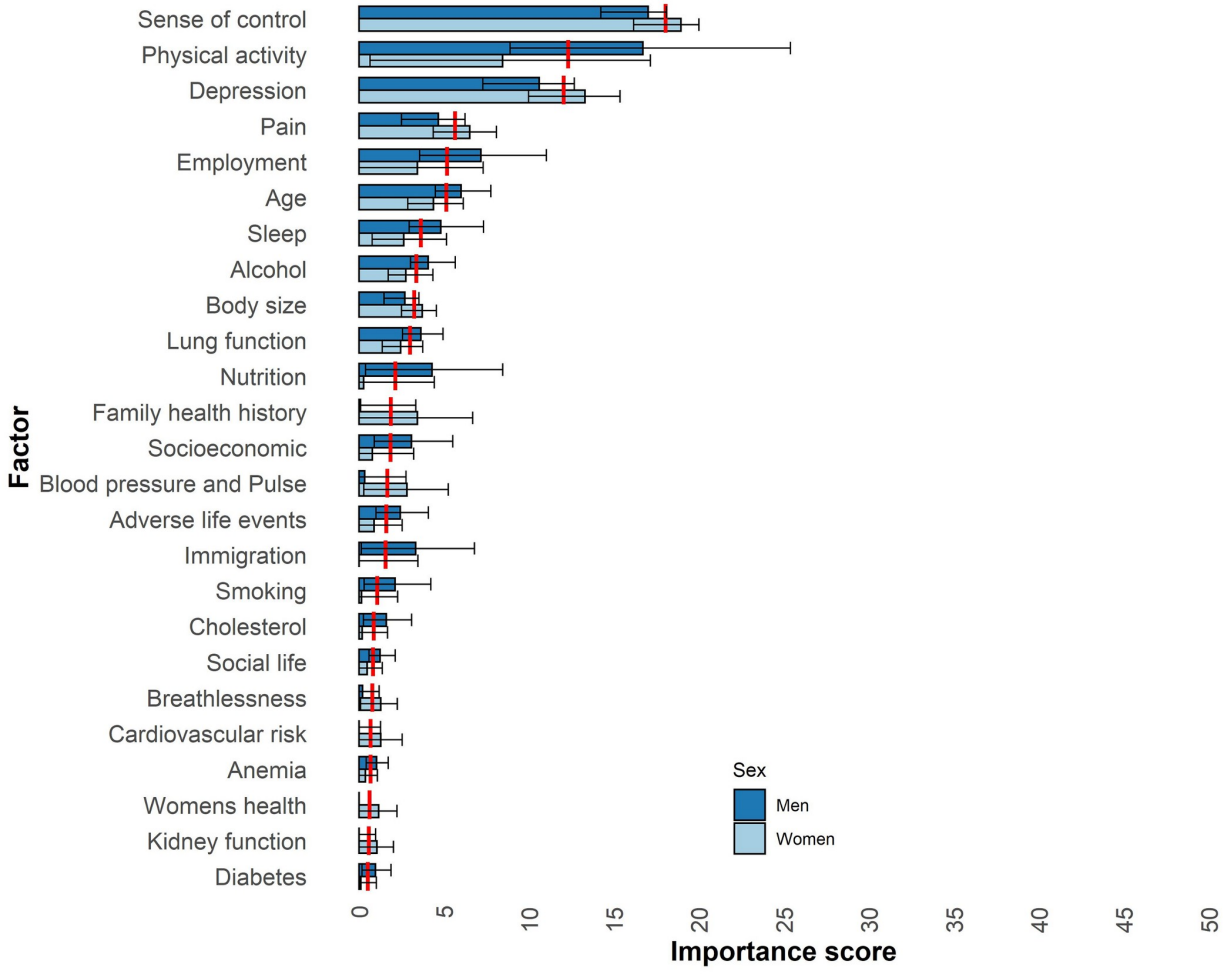
Factors related to mental HRQoL among men (n = 14,124) and women (n = 15,088) aged 50–64 in Sweden. The factors are the summarised importance scores of similar variables, which were assigned before the analysis. The importance score corresponds to the SHapley Additive exPlanations (SHAP) absolute mean divided by the models’ mean absolute error, which is comparable to the effect size. The SHAP absolute mean corresponds to the average degree of change from the mean score of the Short Form 12 for mental health-related quality of life (HRQoL; men: 37.1; women: 37.3) by a predictor variable among all participants. The plot shows the top 25 domains with the highest importance scores for mental HRQoL among all participants and are ordered in descending order. The red line marks the average importance score for all participants. The error bars present the 95% confidence intervals calculated by t-tests.

The factors most important for mental HRQoL among men, women, and all participants are shown in Fig 2 as well as S3 Table. The most important factors for mental HRQoL for all participants (men and women analysed together) were as follows: sense of control (with an importance score of 18), physical activity (12), depression (12), pain (6), employment (5), age (5), sleep (4), and alcohol (3). The order of the factors’ importance for mental HRQoL was different between the sexes. Among men, the following factors were ranked higher in comparison to women (men’s ranking): physical activity (second), employment (fourth), and sleep (sixth). Among women, the following factors were ranked higher than among men (women’s ranking): depression (second), pain (fourth), body size (sixth), and family health history (eighth).

There were some numerical differences between the sexes in the importance scores of the important factors for mental HRQoL. However, none of these differences were statistically significant, as indicated by the overlapping CIs in Fig 2. S4 Table presents the individual variables’ importance scores for physical and mental HRQoL among all participants and separately for men and women.

#### Shape of the associations with physical and mental HRQoL

**Fig 3:**
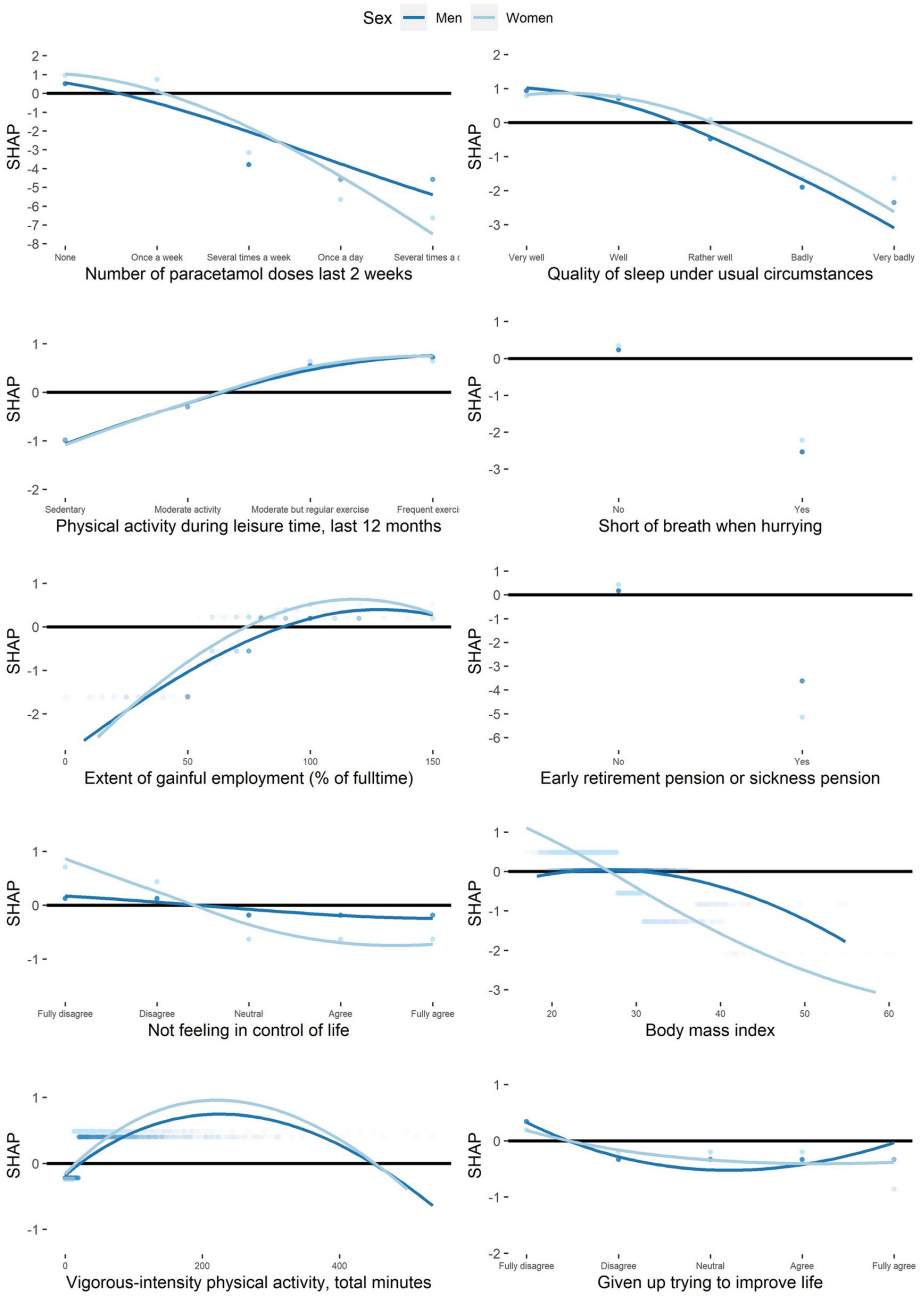
Shapes of associations between variables and physical HRQoL. The scatter plots show the factors with the top ten most important variables for physical health-related quality of life (HRQoL). Participants are represented by dots. The X-axis corresponds to the participants’ values of the variable. The SHapley Additive exPlanations. (SHAP) value (Y-axis) corresponds to the predicted change in physical HRQoL by the model for the participants. The lines are locally estimated scatterplot smoothing lines and can be seen as an average estimation of the predicted physical HRQoL among all participants. Variables without lines are dichotomous. The black horizontal line marks zero. Vigorous-intensity physical activity, total minutes corresponds to total minutes per week.

Fig 3 presents the shapes of the associations for the top ten most important individual variables for physical HRQoL among men and women separately. Increased pain (indicated by higher paracetamol intake), less self-reported physical activity, shortness of breath when hurrying, less gainful employment, and giving up trying to improve life were all associated with worse physical HRQoL among both sexes. Physical activity measured by accelerometry (total minutes per week) had a bell-shaped association with physical HRQoL, with a peak at approximately 200 minutes weekly.

**Fig 4:**
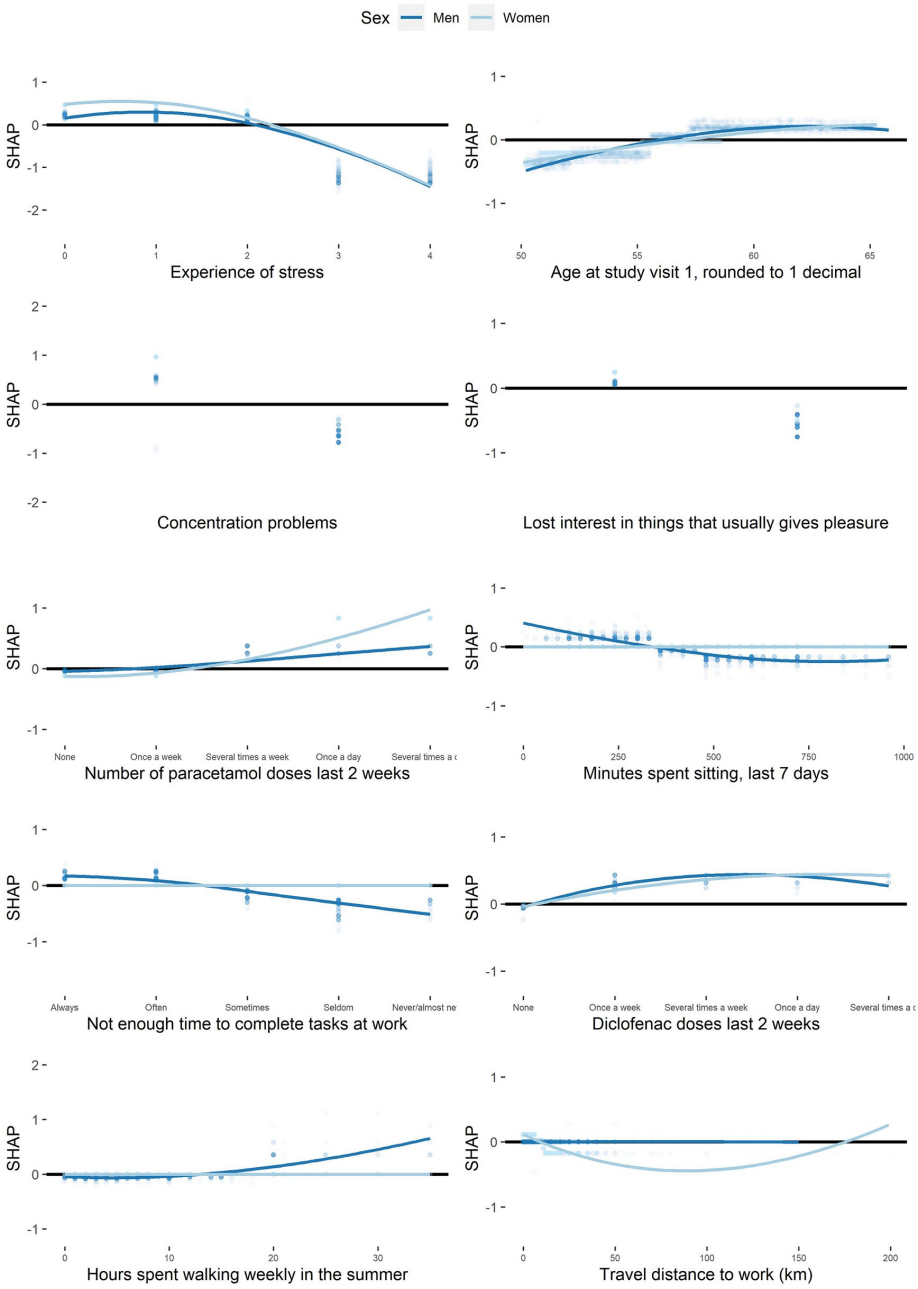
Shapes of associations between variables and mental HRQoL. The scatter plots show the factors with the top ten most important variables for mental HrQoL. Participants are presented by dots. The X-axis corresponds to the participants’ values of the variable. The SHapley Additive exPlanations. (SHAP) value (Y-axis) corresponds to the predicted change in mental health-related quality of life (HRQoL) by the model for the participants. The lines are locally estimated scatterplot smoothing lines and can be seen as an average estimation of the predicted mental HRQoL among all participants. The black horizontal line represents zero. Experience of stress categories: 0 = Never; 1 = Some periods; 2 = Several periods last 5 years; 3 = Permanent stress last year; 4 = Permanent stress last 5 years.

Fig 4 presents the shapes of the associations for the top ten most important individual factors for mental HRQoL among men and women. An increased experience of stress had a dose‒response association with decreased mental HRQoL among both sexes. There was a dose‒response of increased pain (indicated by higher intake of paracetamol and diclofenac) and better HRQoL.

## Discussion

In this large general population study, middle-aged men and women had similar levels of physical and mental HRQoL. The factors most strongly related to HRQoL were physical activity, employment, pain, and sense of control; these findings applied to both physical and mental HRQoL across both sexes. However, some factors seem to be particularly important for men’s and women’s HRQoL: pain was more important for physical and mental HRQoL among women than among men, and physical activity was more important for mental HRQoL among men than among women.

The present findings are novel in several ways. First, the findings that men and women had overall similar HRQoL differed from previous reports of worse HRQoL in women [8, 9]. This may be explained by the overall larger sample size in the present study, which reduced the risk of random differences between the sexes, as well as Sweden’s relatively high socioeconomic gender equality, as indicated by the characteristics of the present study`s participants. Socioeconomic status is a strong predictor of health and is far from equal between the sexes in many countries worldwide, where socioeconomic status may be a more important factor for HRQOL [6]. A second novel finding is that most of the factors were similarly associated with HRQoL between the sexes, and with only a few exceptions, we believe that public health interventions targeting both men and women can be beneficial for overall public health.

Physical activity was identified as the most important factor for both physical and mental HRQoL and corresponded to a large proportion of the differences in physical and mental HRQoL. The health benefits of physical activity are well documented [23]. Reinforced by the shape of association plots, our study shows that 200 minutes of vigorous weekly exercise is strongly associated with better HRQoL, which is in line with the WHO recommendations of 75–150 minutes weekly vigorous exercise or 150–300 minutes weekly moderate-intensity exercise [24]. However, resistance exercise of 60 min per week has previously been recommended for healthy ageing and longevity, but for healthy individuals there is no convincing evidence to limit minutes of resistance exercise per week [25].

Pain was very important for physical and mental HRQoL in our study. Pain is associated with multiple adverse health effects; it is the most important factor for self-perceived health [20] and can be described as a silent epidemic [26]. Many older adults also complain that they need stronger pain relieving medications [27]. The explanation of the association between the use of paracetamol and diclofenac and better mental HRQoL is not obvious. One explanation for diclofenac is that the treatment can pose a cardiovascular health risk [28], and risk groups of cardiovascular diseases can use the treatments to a lesser extent, and these participants might possess an overall worse mental HRQoL.

Sense of control was shown to be important for HRQoL in our study and could reflect the effects from characteristics that affect the individual’s life that they feel is out of their reach to improve. This can, for example, be chronic diseases, work situations, and social status–factors that can affect HRQoL and decrease the individual’s independence. We expect that especially marginalised groups with limited capability to change or influence aspects of their lives are affected by sense of control in relation to HRQoL [29].

Employment’s importance for mental HRQoL was especially reflected by work-related stress, support from colleagues, and influence over what to do at work, which can be compared with sense of control. Employment’s importance for physical HRQoL was largely reflected by early retirement and sick leave, which can be attributed to the physical limitations induced by the cause of the individual’s early retirement or sick leave. Influence on decisions at work was also important for physical HRQoL, and we believe that this can be attributed to physically demanding work environments requiring only lower education with low control over decisions.

The strengths of this study include the large sample size and the cohort being representative of the Swedish general population [13], which strengthens the generalisability. The use of machine learning methods enabled the analysis of the interplay between a wide range of factors representing a large spectrum of health aspects present in people’s everyday lives. The SF-12 is widely used to measure HRQoL and has been validated in different settings and populations [14], thereby enabling comparability with other studies. Another widely used HRQoL instrument is the EQ-5D [30]. However, the use of EQ-5D has been shown to present less variance of the HRQoL scores between participants because of the ceiling effect of the EQ-5D [30], meaning that an individual has to be severe sick to show any decreased HRQoL score. This could favour the SF-12 over the EQ-5D as an outcome in general population studies with overall healthy participants. Repeating these analyses with different measures of quality of life will be important, future work. The models used in this study show good generalisability when predicting unseen data from a separate study site, which further strengthens the generalisability of the present study’s findings.

The limitations of the present study include its nonrandomised cross-sectional design, wherefore causality cannot be inferred. However, the causative effects of many of the identified factors, such as physical activity and pain, on HRQoL have been widely studied [23, 26]. Another limitation is that pain was not reported directly by instruments; instead, “pain treated with nonprescription drugs” was used as a proxy for pain. Finally, as our study used a novel approach on an understudied field, it is challenging to compare our results with previous studies due to differences in the assumptions of the analysis methods used.

The present findings have several important implications. Physical activity, pain, and employment were identified as key factors that are likely to be amenable to interventions, and this study can support prioritising interventions. The identified factors need to be a target even before middle age to lay the foundation for long and happy lives. Considering the findings regarding physical activity of our study, previous studies [23, 31], and WHO recommendations [24], we suggest public health interventions promoting more regular physical activity among the whole population. However, future studies should focus on evaluating what type of physical activity would be most beneficial for HRQOL and well-being, especially in people who may already have some physical limitations due to co-morbidities. Our findings on the importance of pain also align with our findings of the importance of physical activity, which can be used as prevention against pain [32], but also against early retirement and sick leave, which were important within the employment factor. With our study’s findings in mind, we suggest a research focus on the prevention of pain in and before (throughout life) in middle age to increase the chances of a life free from pain in later ages. Finally, as many of the factors were similarly important to HRQoL, our findings indicate that a similar set of factors should be targeted in men and women. The potential is great, as healthy lifestyles are unequally distributed in the population.

In conclusion, middle-aged men and women had similar HRQoL, and the factors most strongly related to physical and mental HRQoL are physical activity, employment, pain, and sense of control. All these factors can be modified on an individual level and should be targeted by public health interventions that are aimed towards both men and women overall.

## Supporting information

S1 Table Abbreviations: CABG = coronary artery bypass graft; COPD = Chronic obstructive pulmonary disease; FEV1 = forced expiratory volume in 1 second; FVC = Forced vital capacity; Hb = haemoglobin; HbA1c = haemoglobin A1c; HDL = high-density lipoprotein; hsCRP = High-sensitivity C-reactive protein; IBD = inflammatory bowel disease; LDL = low-density lipoprotein; *MEF50 =* maximal expiratory flow at 50% of the forced vital capacity; MI = myocardial infarction; OLD = obstructive lung disease; PCI = Percutaneous Coronary Intervention; SLE = Systemic lupus erythematosus; VCmax = Maximum vital capacity.
(DOCX)

S2 Table Hyperparameters were selected by 10-folded cross-validation on the training set. The selected hyperparameters were used in the final models that were evaluated on the test set.
(DOCX)

S3 Table The factors are the summarised importance scores of similar variables, which were assigned before the analysis. The importance score corresponds to the SHapley Additive exPlanations (SHAP) absolute mean divided by the models’ mean absolute error, which is comparable to the effect size. The SHAP absolute mean corresponds to the average degree of change from the mean score of the Short Form 12 for physical HrQoL (men: 53.4; women: 51.4) by a predictor variable among all participants. Abbreviations: CABG = coronary artery bypass graft; IBD = inflammatory bowel disease; MI = myocardial infarction; OLD = obstructive lung disease; PCI = Percutaneous Coronary Intervention.
(DOCX)

S4 Table The importance score corresponds to the SHapley Additive exPlanations (SHAP) absolute mean divided by the models’ mean absolute error, which is comparable to the effect size. The SHAP absolute mean corresponds to the average degree of change from the mean score of the Short Form 12 for physical HrQoL (men: 53.4; women: 51.4) by a predictor variable among all participants. Abbreviations: CABG = coronary artery bypass graft; COPD = Chronic obstructive pulmonary disease; FEV1 = forced expiratory volume in 1 second; FVC = Forced vital capacity; Hb = haemoglobin; HbA1c = haemoglobin A1c; HDL = high-density lipoprotein; hsCRP = High-sensitivity C-reactive protein; IBD = inflammatory bowel disease; LDL = low-density lipoprotein; *MEF50 =* maximal expiratory flow at 50% of the forced vital capacity; MI = myocardial infarction; OLD = obstructive lung disease; PCI = Percutaneous Coronary Intervention; SLE = Systemic lupus erythematosus; VCmax = Maximum vital capacity.
(DOCX)

S1 Fig The process was conducted when training four separate models for predicting: Physical HRQoL among men; physical HRQoL among women; mental HRQoL among men; and mental HRQoL among women. The Stockholm study site was used as the test sets. For the men´s physical and mental HRQoL models, the test sets comprised of 18% of the total men participant. For the women´s physical and mental HRQoL models the test sets comprised of 16% of the total women participants.
(JPEG)

S1 Checklist STROBE statement—Checklist of items that should be included in reports of *cross-sectional studies*. (DOCX)
