# Supplementary material for: Factors important for health-related quality of life in men and women: The population based SCAPIS study
Source: PLoS One. 2023 Nov 3;18(11):e0294030. doi: 10.1371/journal.pone.0294030 (PMC10624288; doi:10.1371/journal.pone.0294030)
Supplement: S1 Table — Abbreviations: CABG = coronary artery bypass graft; COPD = Chronic obstructive pulmonary disease; FEV1 = forced expiratory volume in 1 second; FVC = Forced vital capacity; Hb = haemoglobin; HbA1c = haemoglobin A1c; HDL = high-density lipoprotein; hsCRP = High-sensitivity C-reactive protein; IBD = inflammatory bowel disease; LDL = low-density lipoprotein; MEF50 = maximal expiratory flow at 50% of the forced vital capacity; MI = myocardial infarction; OLD = obstructive lung disease; PCI = Percutaneous Coronary Intervention; SLE = Systemic lupus erythematosus; VCmax = Maximum vital capacity. (DOCX) [file pone.0294030.s001.docx]

**S1 Table – Description of variables and factor categorisations in the analysis.**

| **Variable** | **Factor** | **Values** | |
| --- | --- | --- | --- |
| Ability to find 20 000 Swedish krona (SEK) in a week for unforeseen events | Socioeconomic | Yes; No | |
| Accelerometer wear time (minutes per day) | Physical activity | Numeric | |
| Accelerometer wear time (total minutes) | Physical activity | Numeric | |
| Acetylsalicylic acid, last 2 weeks, self-reported | Non-prescription pain killers | None; Once a week; Several times a week; Once a day; Several times a day. | |
| Action taken at pain or discomfort in chest | Chest pain | Slow down; Stop; Continue at the same pace | |
| Age at first menstruation | Women’s health | Numeric | |
| Age at first snus usage occasion | Other nicotine than cigarettes | Numeric | |
| Age at smoke start (derived variable) | Smoking | Numeric | |
| Age at study visit 1, rounded to 1 decimal | Age | Numeric | |
| Allergic rhinitis, last 12 months, self-reported | Allergy | Yes; No | |
| Angina pectoris, doctor-diagnosed, self-reported | Angina | Yes; No | |
| Angina pectoris, medication last 2 weeks, self-reported | Angina | Yes; No | |
| Ankle-brachial index | Blood pressure and Pulse | Numeric | |
| Annoyed someone because of drinking | Alcohol | Yes; No | |
| Aortic intervention, location, self-reported | Aortic intervention | Chest; Abdomen. | |
| Aortic intervention, self-reported | Aortic intervention | Yes; No | |
| Aortic intervention, year of intervention, self-reported | Aortic intervention | Numeric | |
| Approximate weight at 20 years | Body size | Numeric | |
| Arm of doppler measurement, systolic Blood Pressure. | Blood pressure and Pulse | Right; Left. | |
| Asthma, age of onset, self-reported | OLD | Numeric | |
| Asthma, doctor-diagnosed, self-reported | OLD | Yes; No | |
| Asthma, medication last 2 weeks, self-reported | OLD | Yes; No | |
| Atrial fibrillation, doctor-diagnosed, self-reported | Atrial fibrillation | Yes; No | |
| Atrial fibrillation, medication last 2 weeks, self-reported | Atrial fibrillation | Yes; No | |
| Attempts to cut down on drinking | Alcohol | Yes; No | |
| Average daily cigarette consumption during years of smoking (derived variable) | Smoking | Numeric | |
| Average income in neighbourhood | Socioeconomic | Numeric | |
| Average physical activity intensity; Mean Vector Magnitude in counts per minute | Physical activity | Numeric | |
| Bad self-esteem/feeling worthless | Depression | Yes; No | |
| Body height | Body size | Numeric | |
| Body mass index | Body size | Numeric | |
| Body weight | Body size | Numeric | |
| Born in Sweden | Immigration | Yes; No | |
| Breathing problems affecting daily activites | Breathlessness | Yes; No | |
| Breathless when climbing two flights of stairs | Breathlessness | Yes; No | |
| Breathless when walking on level ground | Breathlessness | Yes; No | |
| Breathlessness preventing leaving home | Breathlessness | Yes; No | |
| CABG or PCI intervention, self-reported | CABG or PCI | Yes; No | |
| CABG or PCI, type of intervention, self-reported | CABG or PCI | Coronary artery bypass graft surgery (CABG) ; Percutaneous Coronary Intervention (PCI); CABG and PCI. | |
| CABG or PCI, year of intervention, self-reported | CABG or PCI | Numeric | |
| Calculated nutrition value: alcohol | Alcohol | Numeric | |
| Calculated nutrition value: carbohydrates | Nutrition | Numeric | |
| Calculated nutrition value: cholesterol | Nutrition | Numeric | |
| Calculated nutrition value: energy (kcal) | Nutrition | Numeric | |
| Calculated nutrition value: fat | Nutrition | Numeric | |
| Calculated nutrition value: protein | Nutrition | Numeric | |
| Cancer, doctor-diagnosed, self-reported | Cancer | Yes; No | |
| Cancer, medication last 2 weeks, self-reported | Cancer | Yes; No | |
| Cancer, year of diagnosis (year - yyyy) | Cancer | Numeric | |
| Cardiovascular risk SCORE-value | Cardiovascular risk | |  |
| Celiac disease, doctor-diagnosed, self-reported | Celiac disease | Yes; No | |
| Celiac disease, year of diagnosis, self-reported | Celiac disease | Numeric | |
| Chance of dozing: as a passenger in a car for an hour without a break | Sleep | None; Slight; Moderate; High | |
| Chance of dozing: in a car, while stopped for a few minutes in the traffic | Sleep | None; Slight; Moderate; High | |
| Chance of dozing: lying down to rest in the afternoon when circumstances permit | Sleep | None; Slight; Moderate; High | |
| Chance of dozing: sitting and reading | Sleep | None; Slight; Moderate; High | |
| Chance of dozing: sitting and talking to someone | Sleep | None; Slight; Moderate; High | |
| Chance of dozing: sitting inactive in a public place (e.g. a theatre or a meeting) | Sleep | None; Slight; Moderate; High | |
| Chance of dozing: sitting quietly after a lunch without alcohol | Sleep | None; Slight; Moderate; High | |
| Chance of dozing: watching TV | Sleep | None; Slight; Moderate; High | |
| Cholesterol Numerical result | Cholesterol | Numeric | |
| Chronic bronchitis, doctor-diagnosed, self-reported | Chronic bronchitis | Yes; No | |
| Chronic rhinosinusitis (CRS), doctor-diagnosed, self-reported | Allergy | Yes; No | |
| Classification of chronic airway limitation based on value of FEV1/FVC | Lung function | Chronic airflow limitation; No chronic airflow limitation. | |
| Classification of glycaemic status | Diabetes | Normoglycemia; Impaired fasting glucose; Elevated HbA1c; New diabetes mellitus diagnosis; Previously known diabetes mellitus. | |
| Cohabitants smoking at home | Smoking | No; Yes, less than 10 years; Yes, 10-20 years; Yes, more than 20 years. | |
| Concentration problems | Depression | Yes; No | |
| Concerns expressed by others about drinking | Alcohol | Yes; No | |
| COPD, chronic bronchitis or emphysema, Doctor-diagnosed, self-reported | OLD | Yes; No | |
| COPD, chronic bronchitis or emphysema, medication last 2 weeks, doctor-diagnosed, self-reported | OLD | Yes; No | |
| COPD, doctor-diagnosed, self-reported | OLD | Yes; No | |
| Coronary dominance | Blood pressure and Pulse | Left; Right; Balanced | |
| Coughing most days for at least three months every year | Coughing | Yes; No | |
| Coughing when not having a cold | Coughing | Yes; No | |
| Creatinine Numerical result | Kidney function | Numeric | |
| Crohn's disease or ulcerative colitis, doctor-diagnosed, self-reported | IBD | Yes; No | |
| Crohn's disease or ulcerative colitis, medication, self-reported | IBD | Yes; No | |
| Crohn's disease or ulcerative colitis, year of diagnosis, self-reported | IBD | Numeric | |
| Current smoking status, self-reported | Smoking | Regular; Occasional; Former; Never. | |
| Current snus users: number of snus cans per week | Other nicotine than cigarettes | <2 cans per week;2 to 4 cans per week; 5-6 cans per week; more than 7 cans per week. | |
| Daily usage of other nicotine products | Other nicotine than cigarettes | Yes; No | |
| Daily usage of snus for more than one month | Other nicotine than cigarettes | Ye; No; Former. | |
| Daily use of chewing tobacco | Other nicotine than cigarettes | Yes; No | |
| Daily use of nicotine replacements | Other nicotine than cigarettes | Yes; No | |
| Degree of physical activity at work | Physical activity | Sitting/Standing; Light work; Light mobile work; Sometimes heavy; Heavy work. | |
| Diabetes, age of onset, self-reported | Diabetes | Numeric | |
| Diabetes, doctor-diagnosed, self-reported | Diabetes | Yes; No | |
| Diabetes, medication last 2 weeks, self-reported | Diabetes | Yes; No | |
| Diastolic blood pressure, mean brachial (recommended for general analysis) | Blood pressure and Pulse | Numeric | |
| Diclofenac doses last 2 weeks | Non-prescription pain killers | None; Once a week; Several times a week; Once a day; Several times a day. | |
| Difficulties managing regular expenses, last 12 months | Socioeconomic | Yes; No | |
| Diffusion capacity for carbon monoxide | Lung function | Numeric | |
| Diffusion capacity for carbon monoxide, related to alveolar volume | Lung function | Numeric | |
| Discoloured nasal discharge, >12 weeks during last 12 months, self-reported | Allergy | Yes; No | |
| Divorced | Living together with others | Yes; No | |
| Drinks alcoholic cider and soda | Alcohol | Yes; No | |
| Drinks beer | Alcohol | Yes; No | |
| Drinks hard liquor (incl. drinks, whiskey, liqueur etc.) | Alcohol | Yes; No | |
| Drinks wine | Alcohol | Yes; No | |
| Early retirement pension or sickness pension | Employment | Yes; No | |
| Emphysema, doctor-diagnosed, self-reported | Emphysema | Yes; No | |
| Ever had severe pain in chest >30 min | Chest pain | Yes; No | |
| Expectance of mostly positive experiences next 5-10 years | Sense of control | Fully disagree; Disagree; Neutral; Agree; Fully agree. | |
| Experience of stress | Sense of control | Never; Some periods; Some period last year; Perpetual last year; Perpetual last 5 years. | |
| Ex-smokers: average grams of pipe tobacco per day - exsmoker | Smoking | Numeric | |
| Ex-smokers: average number of cigarettes per day | Smoking | Numeric | |
| Ex-smokers: average number of cigars/cigar-cigarettes per day | Smoking | Numeric | |
| Extent of gainful employment (% of fulltime) | Employment | Numeric | |
| Facial pain or pressure, >12 weeks during last 12 months, self-reported | Allergy | Yes; No | |
| Family history of asthma, any first degree relative | Family health history | Yes; No | |
| Family history of asthma, subject's children | Family health history | Yes; No | |
| Family history of asthma, subject's father | Family health history | Yes; No | |
| Family history of asthma, subject's mother | Family health history | Yes; No | |
| Family history of asthma, subject's sibling | Family health history | Yes; No | |
| Family history of bronchitis, COPD or emphysema, any first degree relative | Family health history | Yes; No | |
| Family history of bronchitis, COPD or emphysema, subjects children | Family health history | Yes; No | |
| Family history of bronchitis, COPD or emphysema, subjects father | Family health history | Yes; No | |
| Family history of bronchitis, COPD or emphysema, subjects mother | Family health history | Yes; No | |
| Family history of bronchitis, COPD or emphysema, subjects siblings | Family health history | Yes; No | |
| Family history of diabetes, any first degree relative | Family health history | Yes; No | |
| Family history of diabetes, subject's children | Family health history | Yes; No | |
| Family history of diabetes, subject's father | Family health history | Yes; No | |
| Family history of diabetes, subject's mother | Family health history | Yes; No | |
| Family history of diabetes, subject's sibling | Family health history | Yes; No | |
| Family history of lung cancer, subject's father | Family health history | Yes; No | |
| Family history of lung cancer, subject's mother | Family health history | Yes; No | |
| Family history of lung cancer, subject's parent or sibling | Family health history | Yes; No | |
| Family history of lung cancer, subject's sibling | Family health history | Yes; No | |
| Family history of myocardial infarction, subject's father | Family health history | Yes; No | |
| Family history of myocardial infarction, subject's mother | Family health history | Yes; No | |
| Family history of myocardial infarction, subject's parent or sibling | Family health history | Yes; No | |
| Family history of myocardial infarction, subject's sibling | Family health history | Yes; No | |
| Family history of stroke, subject's father | Family health history | Yes; No | |
| Family history of stroke, subject's mother | Family health history | Yes; No | |
| Family history of stroke, subject's parent or sibling | Family health history | Yes; No | |
| Family history of stroke, subject's sibling | Family health history | Yes; No | |
| Father's biological figure at 40 years of age | Family health history | Nine different figures to choose from. | |
| Feeling of being unfairly treated | Sense of control | Fully disagree; Disagree; Neutral; Agree; Fully agree. | |
| Feelings of sadness/depression, last 12 months | Depression | Yes, past year; Previously but not past year; No. | |
| Felt guilt about drinking | Alcohol | Yes; No | |
| Felt tired or low on energy | Depression | Yes; No | |
| FEV1/FVC post bronchodilatation | Lung function | Numeric | |
| FEV1/VCmax post bronchodilatation | Lung function | Numeric | |
| Forced expiratory volume in one second post-bronchodilation | Lung function | Numeric | |
| Forced vital capacity (FVC) post bronchodilation. | Lung function | Numeric | |
| Former snus user: number of snus cans per week | Other nicotine than cigarettes | <2 cans per week;2 to 4 cans per week; 5-6 cans per week; more than 7 cans per week. | |
| Frequency drinking resulting in blackouts, last year | Alcohol | Less than monthly; Monthly; Weekly; Daily or almost daily | |
| Frequency failing to do what is expected due to drinking, last year | Alcohol | Less than monthly; Monthly; Weekly; Daily or almost daily | |
| Frequency feeling guilty about drinking, last year | Alcohol | Less than monthly; Monthly; Weekly; Daily or almost daily | |
| Frequency having >6 alcoholic drinks on one occasion | Alcohol | Less than monthly; Monthly; Weekly; Daily or almost daily | |
| Frequency having an alcoholic drink, last year | Alcohol | Never; Monthly or less; 2 to 4 times a month; 2 to 3 times a week; 4 or more times a week; | |
| Frequency needing a drink in the morning after drinking the night before, last year | Alcohol | Less than monthly; Monthly; Weekly; Daily or almost daily | |
| Frequency not being able to stop drinking, last year | Alcohol | Less than monthly; Monthly; Weekly; Daily or almost daily | |
| Frequency of difficulty to fall asleep at night | Sleep | Never, < 1 week; 1-2 times a week; 3-6 week; Almost every night. | |
| Frequency of loud snoring (according to self or others) | Sleep | Never; Seldom; Sometimes; Frequently; Very frequently. | |
| Frequency of reflux after going to bed | Sleep | Never, < 1 week; 1-2 times a week; 3-6 week; Almost every night. | |
| Frequency of waking up several times during the night | Sleep | Never, < 1 week; 1-2 times a week; 3-6 week; Almost every night. | |
| Frequency of waking up too early without being able to go back to sleep | Sleep | Never, < 1 week; 1-2 times a week; 3-6 week; Almost every night. | |
| Gained or lost weight | Depression | Yes; No | |
| Gestational diabetes, self-reported | Women’s health | Yes; No; Have not been pregnant; | |
| Given up trying to improve life | Sense of control | Fully disagree; Disagree; Neutral; Agree; Fully agree. | |
| Glucose, capillary, day 1 | Diabetes | Numeric | |
| Glucose, venous, day 1, numerical result | Diabetes | Numeric | |
| Hb Numerical Result | Anemia | Numeric | |
| HbA1c Numerical result | Diabetes | Numeric | |
| HDL Numerical result - national variable. | Cholesterol | Numeric | |
| Heart failure, medication last 2 weeks, self-reported | Heart failure | Yes; No | |
| Heart failure, self-reported, doctor-diagnosed | Heart failure | Yes; No | |
| Heart valve disease, doctor-diagnosed, self-reported | Heart valve disease | Yes; No | |
| Heart valve disease, type, self-reported | Heart valve disease | Aortic valve; more than one valve; Mitral valve; Other Valve. | |
| Heart valve disease, year of intervention, self-reported | Heart valve disease | Numeric | |
| High degree of unexpected changes in life, past 10 years | Sense of control | Fully disagree; Disagree; Neutral; Agree; Fully agree. | |
| Highest completed level of education | Education | University; upper secondary school; elementary school; Not completed elementary school. | |
| Hip circumference | Body size | Numeric | |
| Hormone treatment of menopausal symptoms, self-reported | Women’s health | Yes; No | |
| Hours bicycling: winter | Physical activity | Numeric | |
| Hours of sleep per night under usual circumstances | Sleep | 4 hours or less; 5 hours; 6 hours; 7 hours; 8 hours; 9 hours; 10 hours or more; | |
| Hours spent bicycling: summer | Physical activity | Numeric | |
| Hours spent walking: summer | Physical activity | Numeric | |
| Hours spent walking: winter | Physical activity | Numeric | |
| hsCRP Numerical result - national variable | Inflammation | Numeric | |
| Hyperlipidaemia, doctor-diagnosed, self-reported | Cholesterol | Yes; No | |
| Hyperlipidaemia, medication last 2 weeks, self-reported | Cholesterol | Yes; No | |
| Hypertension, doctor-diagnosed, self-reported | Hypertension | Yes; No | |
| Hypertension, medication last 2 weeks, self-reported | Hypertension | Yes; No | |
| If you did not have a period, what was the reason? Response alternative: medication. | Women’s health | Yes; No | |
| Injuries caused by drinking | Alcohol | Yes; No | |
| LDL Numerical result - national variable. | Cholesterol | Numeric | |
| Life events: concerns for someone close | Life events | Yes, past year; Previously but not past year; No. | |
| Life events: death of someone close | Life events | Yes, past year; Previously but not past year; No. | |
| Life events: felt insecure at work | Life events | Yes, past year; Previously but not past year; No. | |
| Life events: had to change housing | Life events | Yes, past year; Previously but not past year; No. | |
| Life events: had to change job | Life events | Yes, past year; Previously but not past year; No. | |
| Life events: loss of job | Life events | Yes, past year; Previously but not past year; No. | |
| Life events: own divorce of separation | Life events | Yes, past year; Previously but not past year; No. | |
| Life events: received criminal penalty | Life events | Yes, past year; Previously but not past year; No. | |
| Life events: serious financial problems | Life events | Yes, past year; Previously but not past year; No. | |
| Life events: serious illness/accident in family | Life events | Yes, past year; Previously but not past year; No. | |
| Living Alone | Living together with others | Yes; No | |
| Living in own apartment | Socioeconomic | Yes; No | |
| Living in rental appartment | Socioeconomic | Yes; No | |
| Living in villa | Socioeconomic | Yes; No | |
| Sense of control: at work | Sense of control | Fully disagree; Disagree; Neutral; Agree; Fully agree. | |
| Lost interest in things that usually gives pleasure | Depression | Yes; No | |
| Low-intensity physical activity (LIPA), average minutes per day | Physical activity | Numeric | |
| Low-intensity physical activity (LIPA), percentage of wear time | Physical activity | Numeric | |
| Low-intensity physical activity (LIPA), total minutes | Physical activity | Numeric | |
| Lung disease (other than COPD, chronic bronchitis or emphysema), doctor diagnosed, self-reported | Other lung disease | Yes; No | |
| Married | Living together with others | Yes; No | |
| MEF50 post bronchodilatation | Lung function | Numeric | |
| Menstruation, last year | Women’s health | Yes; No | |
| Minutes spent sitting, last 7 days | Physical activity | Numeric | |
| Mode of travel to work: autumn | Physical activity | Bicycling; Car; Walking; Bus/tram/train. | |
| Mode of travel to work: spring | Physical activity | Bicycling; Car; Walking; Bus/tram/train. | |
| Mode of travel to work: summer | Physical activity | Bicycling; Car; Walking; Bus/tram/train. | |
| Mode of travel to work: winter | Physical activity | Bicycling; Car; Walking; Bus/tram/train. | |
| Moderate- and vigorous-intensity physical activity (MVPA), average minutes per day | Physical activity | Numeric | |
| Moderate- and vigorous-intensity physical activity (MVPA), percentage of wear time | Physical activity | Numeric | |
| Moderate- and vigorous-intensity physical activity (MVPA), total minutes | Physical activity | Numeric | |
| Moderate intensity physical activity (MPA), total minutes | Physical activity | Numeric | |
| Moderate-intensity physical activity (MPA), average minutes per day | Physical activity | Numeric | |
| Moderate-intensity physical activity (MPA), percentage of wear time | Physical activity | Numeric | |
| Month of accelerometer recording | Physical activity | Numeric | |
| Mother's biological figure at 40 years of age | Family health history | Nine different figures to choose from. | |
| Myocardial infarction, doctor-diagnosed, self-reported | MI | Yes; No | |
| Myocardial infarction, year of first event, self-reported | MI | Numeric | |
| Myocardial infarction, year of latest event, self-reported | MI | Numeric | |
| Nasal Obstruction, >12 weeks during last 12 months, self-reported | Allergy | Yes; No | |
| Need to stop for breath after 100 meters | Breathlessness | Yes; No | |
| Need to stop for breath when walking on level ground | Breathlessness | Yes; No | |
| Non-steroidal anti-inflammatory drugs (NSAID) doses last 2 weeks | Non-prescription pain killers | None; Once a week; Several times a week; Once a day; Several times a day. | |
| Not enough time to complete tasks at work | Employment | Always; often; sometimes; seldom; never. | |
| Not feeling in control of life | Sense of control | Fully disagree; Disagree; Neutral; Agree; Fully agree. | |
| Number of alcoholic cider bottle/cans (>4.5 vol% alcohol) consumed during an average month | Alcohol | 1-3 per month; 4-6 per month; 7-9 per month; 10-12 per month; 13-15 per month; 16-18 per month; | |
| Number of alcoholic cider/soda bottles/cans consumed during an average month | Alcohol | 1-3 per month; 4-6 per month; 7-9 per month; 10-12 per month; 13-15 per month; 16-18 per month; | |
| Number of alcoholic drinks on a typical drinking day | Alcohol | One to two; three to four; five to six; seven to nine; ten or more | |
| Number of days hospitalized due to breathing problems, last 12 months | Breathlessness | Numeric | |
| Number of episdodes with breathing problems requiring health care, last 12 months | Breathlessness | Numeric | |
| Number of episdodes with breathing problems requiring hospitalization, last 12 months | Breathlessness | Numeric | |
| Number of episdodes with breathing problems, last 12 months | Breathlessness | Numeric | |
| Number of extra strong beer bottles/cans (approx >5.2 vol% alcohol) consumed during an average month | Alcohol | 1-3 per month; 4-6 per month; 7-9 per month; 10-12 per month; 13-15 per month; 16-18 per month; | |
| Number of medium-strong beer bottles/cans (2.26-3.5 vol% alcohol) consumed during an average month | Alcohol | 1-3 per month; 4-6 per month; 7-9 per month; 10-12 per month; 13-15 per month; 16-18 per month. | |
| Number of medium-strong beer bottles/cans (approx 3.5-4.5 vol% alcohol) consumed during an average month | Alcohol | 1-3 per month; 4-6 per month; 7-9 per month; 10-12 per month; 13-15 per month; 16-18 per month; | |
| Number of occasions of physical exercise, last 3 months | Physical activity | More than three times per week; 2-3 times a week; 1-2 times a week; Occasionally - not regularly; Never. | |
| Number of paracetamol doses last 2 weeks | Non-prescription pain killers | None; Once a week; Several times a week; Once a day; Several times a day. | |
| Number of strong beer bottles/cans (approx 4.5-5.2 vol% alcohol) consumed during an average month | Alcohol | 1-3 per month; 4-6 per month; 7-9 per month; 10-12 per month; 13-15 per month; 16-18 per month; | |
| Number of valid days of accelerometer recording | Physical activity | Numeric | |
| Number of weekend days of accelerometer recording | Physical activity | Numeric | |
| Number of wine bottles consumed during an average month | Alcohol | <= 0,5 ; 1 ; 2 ; 3 ; 4 ; 5; 6; 7 ; 8; 9 ; >=10 ; | |
| Number of years of smoking (derived variable) | Smoking | Numeric | |
| Other type of living | Socioeconomic | Yes; No | |
| Pack years for cigarettes (derived variable) | Smoking | Numeric | |
| Pain in calves when hurrying or walking uphill | Claudication | Yes; No | |
| Pain or discomfort in chest when hurrying or walking uphill | Chest pain | Yes; No | |
| Pain or discomfort in chest when walking on level ground | Chest pain | Yes; No | |
| Parental heredity for myocardial infarction | Family health history | Yes; No | |
| Parental heredity for stroke | Family health history | Yes; No | |
| Percentage of weekend days of accelerometer recording | Physical activity | Numeric | |
| Peripheral artery disease, intervention, self-reported | Peripheral artery disease | Yes; No | |
| Peripheral artery disease, type of intervention, self-reported | Peripheral artery disease | Coronary artery bypass graft surgery (CABG); Percutaneous Coronary Intervention (PCI); CABG and PCI. | |
| Peripheral artery disease, year of intervention, self-reported | Peripheral artery disease | Numeric | |
| Phlegm problems >3 months per year | Coughing | Yes; No | |
| Phlegm problems when not having a cold | Coughing | Yes; No | |
| Physical activity during leisure time, last 12 months | Physical activity | Sedentary, Moderate activity, Moderate but regular exercise, Frequent exercise. | |
| Polycystic ovary syndrome, self-reported | Women’s health | Yes; No | |
| Percentage unemployed in neighbourhood | Socioeconomic | Numeric | |
| Percentage with financial aid in neighbourhood | Socioeconomic | Numeric | |
| Percentage with foreign background in neighbourhood | Socioeconomic | Numeric | |
| Percentage with low economical status in neighbourhood | Socioeconomic | Numeric | |
| Percentage with university degree in neighbourhood | Socioeconomic | Numeric | |
| Presence of any carotid artery plaque | Stroke | No plaque in either carotid artery; Plaque in both carotid arteries; Plaque in one carotid artery. | |
| Present occupation: Contractual pension | Employment | Yes; No | |
| Present occupation: Gainfully employed | Employment | Yes; No | |
| Present occupation: Labour market measures | Employment | Yes; No | |
| Present occupation: Leave of absence or parental leave | Employment | Yes; No | |
| Present occupation: Long term sick listed (more than 3 months) | Employment | Yes; No | |
| Present occupation: Old age pensioner | Employment | Yes; No | |
| Present occupation: Other - not gainfully employed | Employment | Yes; No | |
| Present occupation: Studying or training | Employment | Yes; No | |
| Present occupation: Unemployed | Employment | Yes; No | |
| Professional work, last 12 months | Employment | Yes; No | |
| Prolonged sedentary (prolonged SED), average minutes per day | Physical activity | Numeric | |
| Prolonged sedentary (prolonged SED), total minutes | Physical activity | Numeric | |
| Pulse rate | Blood pressure and Pulse | Numeric | |
| Quality of sleep under usual circumstances | Sleep | Very well; Well; Rather well; Badly; Very badly. | |
| Quincke edema, self-reported | Allergy | Yes; No | |
| Reason for not having menstruation: menopause | Women’s health | Yes; No | |
| Reduced sense of smell, >12 weeks during last 12 months, self-reported | Allergy | Yes; No | |
| Regular/occasional smokers: average grams of pipe tobacco per day during years of smoking | Smoking | Numeric | |
| Regular/occasional smokers: average number of cigarettes per day during years of smoking | Smoking | Numeric | |
| Regular/occasional smokers: average number of cigars/cigarr-cigarettes per day during years of smoking | Smoking | Numeric | |
| Regular/occasional smokers: grams of pipe tobacco per day presently | Smoking | Numeric | |
| Regular/occasional smokers: number of cigarettes per day presently | Smoking | Numeric | |
| Regular/occasional smokers: number of cigars/cigar-cigarettes per day presently | Smoking | Numeric | |
| Rheumatic disease (e.g. rheumatoid arthritis, Bechterew's disease, psoriatic arthritis, SLE, Sjögren's syndrome), doctor-diagnosed, self-reported | Rheumatic disease | Yes; No | |
| Rheumatic disease (e.g. rheumatoid arthritis, Bechterews disease, psoriatic arthritis, SLE, Sjögren's syndrome), medication last 2 weeks, self-reported | Rheumatic disease | Yes; No | |
| Rheumatic disease (e.g. rheumatoid arthritis, Bechterews disease, psoriatic arthritis, SLE, Sjögren's syndrome), year of diagnosis, self-reported | Rheumatic disease | Numeric | |
| Sedentary (SED), average minutes per day | Physical activity | Numeric | |
| Sedentary (SED), percentage of wear time | Physical activity | Numeric | |
| Sedentary (SED), total minutes | Physical activity | Numeric | |
| Seep Apnoea, doctor-diagnosed, self-reported | Apnoea | Yes; No | |
| Sharing household with: children | Living together with others | Yes; No | |
| Sharing household with: no one | Living together with others | Yes; No | |
| Sharing household with: other adults | Living together with others | Yes; No | |
| Sharing household with: parents/siblings | Living together with others | Yes; No | |
| Sharing household with: spouse/partner | Living together with others | Yes; No | |
| Short of breath when hurrying | Breathlessness | Yes; No | |
| Sleep Apnoea or breathing problems during sleep (according to self or others) | Apnoea | Yes=Ja (Yes) \| Do not know=Vet ej (Do not know) \| NO_ANSWER=Vill/kan ej svara (Not able/willing to reply) \| No=Nej (No) | |
| Sleep Apnoea, year of diagnosis, self-reported | Apnoea | Numeric | |
| Slow vital capacity (SVC) post bronchodilation. | Lung function | Numeric | |
| Social attachment: existence of person for comfort | Social life | Yes; No | |
| Social attachment: existence of person to share feelings of happiness | Social life | Yes; No | |
| Social interaction: number of people in household | Social life | Numeric | |
| Social interaction: number of people met during an ordinary week | Social life | -99=Vill/kan ej svara (Not able/willing to reply) \| -98=Ej applicerbart (Not applicable) \| 0=Ingen (None) \| 1=1-2 \| 2=3-5 \| 3=6-10 \| 4=11-15 \| 5=>15 | |
| Social interaction: number of people who can be easily asked for assistance | Social life | -99=Vill/kan ej svara (Not able/willing to reply) \| -98=Ej applicerbart (Not applicable) \| 0=Ingen (None) \| 1=1-2 \| 2=3-5 \| 3=6-10 \| 4=11-15 \| 5=>15 | |
| Social interaction: number of people who can be turned to in difficulties | Social life | -99=Vill/kan ej svara (Not able/willing to reply) \| -98=Ej applicerbart (Not applicable) \| 0=Ingen (None) \| 1=1-2 \| 2=3-5 \| 3=6-10 \| 4=11-15 \| 5=>15 | |
| Started to work in current profession (year) | Employment | Numeric | |
| Stroke, doctor-diagnosed, self-reported | Stroke | Yes; No | |
| Stroke, year of first event, self-reported | Stroke | Numeric | |
| Stroke, year of latest event, self-reported | Stroke | Numeric | |
| Systolic blood pressure | Blood pressure and Pulse | Numeric | |
| Taking a drink in the morning after a drinking session | Alcohol | Yes; No | |
| TG Numerical result - national variable. | Cholesterol | Numeric | |
| Thoughts about death | Depression | Yes; No | |
| Time for pain or discomfort in chest to disappear | Chest pain | Immediatly, less than ten minuts; after more than 10 minuter; pain is present after a long while; | |
| Travel distance to work (km) | Physical activity | Numeric | |
| Trouble falling asleep | Depression | Yes; No | |
| Tuberculosis, doctor-diagnosed, self-reported | Tuberculosis | Yes; No | |
| Tuberculosis, year of diagnosis, self-reported | Tuberculosis | Numeric | |
| Urticaria, self-reported | Allergy | Yes; No | |
| Vigorous-intensity physical activity, average minutes per day | Physical activity | Numeric | |
| Vigorous-intensity physical activity, percentage of wear time | Physical activity | Numeric | |
| Vigorous-intensity physical activity, total minutes | Physical activity | Numeric | |
| Volume of hard liquor in a usual drink | Alcohol | 4 cl; 6 cl; 8 cl; 10 cl; 12 cl; | |
| Waist circumference | Body size | Numeric | |
| Waist-Hip Ratio | Body size | Numeric | |
| Whistling and wheezing problems in the chest | OLD | Yes; No | |
| Whistling and wheezing problems in the chest, last 12 months | OLD | Yes; No | |
| Whistling and wheezing problems in the chest, only when having a cold, last 12 months | OLD | Yes; No | |
| Whistling, wheezing at the same time as feeling short of breath, last 12 months | OLD | Yes; No | |
| Widow | Living together with others | Yes; No | |
| Work situation: emotionally demanding | Employment | Always; often; sometimes; seldom; never. | |
| Work situation: emotionally involving | Employment | Always; often; sometimes; seldom; never. | |
| Work situation: fast work pace | Employment | Always; often; sometimes; seldom; never. | |
| Work situation: influence over decisions | Employment | Always; often; sometimes; seldom; never. | |
| Work situation: influence over what you do | Employment | Always; often; sometimes; seldom; never. | |
| Work situation: influence over work load | Employment | Always; often; sometimes; seldom; never. | |
| Work situation: support from closest superior | Employment | Always; often; sometimes; seldom; never. | |
| Work situation: support from colleagues | Employment | Always; often; sometimes; seldom; never. | |
| Work situation: workload unevenly distributed | Employment | Always; often; sometimes; seldom; never. | |
| Year of immigration to Sweden | Immigration | Numeric | |
| Year of smoke stop (derived variable) | Smoking | Numeric | |
| Years of coughing | Coughing | Less than 2 years; 2-5 years; more than 5 years | |
| Years of regular dwelling at indoor workplaces were people smoke | Smoking | None; Yes, less than 10 years; Yes, 10-20 years; Yes, more than 20 years. | |
| Years of snus usage | Other nicotine than cigarettes | Numeric | |
| Years with phlegm problems | Coughing | Less than 2 years; 2-5 years; more than 5 years | |

Abbreviations: CABG = coronary artery bypass graft; COPD = Chronic obstructive pulmonary disease; FEV1 = forced expiratory volume in 1 second; FVC = Forced vital capacity; Hb = haemoglobin; HbA1c = haemoglobin A1c; HDL = high-density lipoprotein; hsCRP = High-sensitivity C-reactive protein; IBD = inflammatory bowel disease; LDL = low-density lipoprotein; MEF50 = maximal expiratory flow at 50 % of the forced vital capacity; MI = myocardial infarction; OLD = obstructive lung disease; PCI = Percutaneous Coronary Intervention; SLE = Systemic lupus erythematosus; VCmax = Maximum vital capacity.
