# Supplementary material for: Factors important for health-related quality of life in men and women: The population based SCAPIS study
Source: PLoS One. 2023 Nov 3;18(11):e0294030. doi: 10.1371/journal.pone.0294030 (PMC10624288; doi:10.1371/journal.pone.0294030)
Supplement: S2 Table — The selected hyperparameters were used in the final models that were evaluated on the test set. (DOCX) [file pone.0294030.s002.docx]

**S2 Table – Hyperparameters used in final models.**

|  |  | **Physical HrQoL** | | **Mental HrQoL** | |
| --- | --- | --- | --- | --- | --- |
| **Hyperparameter** | **Explanation** | **Men´s model** | **Women´s model** | **Men´s model** | **Women´s model** |
| nrounds | Number of rounds | 150 | 150 | 50 | 100 |
| max_depth | Maximum tree depth | 1 | 1 | 2 | 1 |
| eta | Learning rate | 0.3 | 0.3 | 0.3 | 0.3 |
| gamma | Minimum loss reduction to create a new leaf node of the tree | 0 | 0 | 0 | 0 |
| colsample_bytree | Subsample ratio for each tree | 0.6 | 0.8 | 0.8 | 0.8 |
| min_child_weight | Minimum sum of instance weight to continue building process | 1 | 1 | 1 | 1 |
| subsample | Subsample ratio of training data | 1 | 1 | 1 | 1 |

Hyperparameters were selected by 10-folded cross-validation on the training set. The selected hyperparameters were used in the final models that were evaluated on the test set.
