# Supplementary material for: Factors important for health-related quality of life in men and women: The population based SCAPIS study
Source: PLoS One. 2023 Nov 3;18(11):e0294030. doi: 10.1371/journal.pone.0294030 (PMC10624288; doi:10.1371/journal.pone.0294030)
Supplement: S3 Table — The importance score corresponds to the SHapley Additive exPlanations (SHAP) absolute mean divided by the models’ mean absolute error, which is comparable to the effect size. The SHAP absolute mean corresponds to the average degree of change from the mean score of the Short Form 12 for physical HrQoL (men: 53.4; women: 51.4) by a predictor variable among all participants. Abbreviations: CABG = coronary artery bypass graft; IBD = inflammatory bowel disease; MI = myocardial infarction; OLD = obstructive lung disease; PCI = Percutaneous Coronary Intervention. (DOCX) [file pone.0294030.s003.docx]

**S3 Table– Factors’ importance score for physical and mental HrQoL among all participants, men, and women.**

|  |  | **Physical HRQoL** |  |  | **Mental HRQoL** |  |
| --- | --- | --- | --- | --- | --- | --- |
| **Factor** | **All participants** | **Men** | **Women** | **All participants** | **Men** | **Women** |
| Age | 1.1 | 0 | 2.01 | 5.13 | 6 | 4.36 |
| Alcohol | 4.21 | 3.85 | 4.5 | 3.36 | 4.06 | 2.74 |
| Allergy | 10.26 | 8.59 | 11.64 | 0.33 | 0 | 0.62 |
| Anaemia | 0.27 | 0.52 | 0.06 | 0.68 | 1.02 | 0.37 |
| Angina | 0 | 0 | 0 | 0 | 0 | 0 |
| Aortic intervention | 0 | 0 | 0 | 0.04 | 0.09 | 0 |
| Apnoea | 3.27 | 1.15 | 5.02 | 0.18 | 0.38 | 0 |
| Atrial fibrillation | 0.14 | 0.31 | 0 | 0 | 0 | 0 |
| Blood pressure and Pulse | 0.76 | 1.62 | 0.05 | 1.65 | 0.33 | 2.81 |
| Body size | 14.04 | 12.66 | 15.17 | 3.24 | 2.69 | 3.72 |
| Breathlessness | 17.73 | 16.39 | 18.84 | 0.77 | 0.2 | 1.27 |
| CABG or PCI | 0 | 0 | 0 | 0 | 0 | 0 |
| Cancer | 0.03 | 0.07 | 0 | 0 | 0 | 0 |
| Cardiovascular risk | 0 | 0 | 0 | 0.68 | 0 | 1.28 |
| Celiac disease | 0.11 | 0.24 | 0 | 0.02 | 0.04 | 0 |
| Chest pain | 2.15 | 2.78 | 1.63 | 0 | 0 | 0 |
| Cholesterol | 0.52 | 1.13 | 0.02 | 0.85 | 1.61 | 0.19 |
| Chronic bronchitis | 0 | 0 | 0 | 0 | 0 | 0 |
| Claudication | 0 | 0 | 0 | 0.07 | 0.15 | 0 |
| Coughing | 3.5 | 5.95 | 1.48 | 0 | 0 | 0 |
| Depression | 6.46 | 4.12 | 8.39 | 12.03 | 10.6 | 13.28 |
| Diabetes | 0.94 | 1.5 | 0.47 | 0.5 | 0.95 | 0.1 |
| Education | 0 | 0 | 0 | 0 | 0 | 0 |
| Emphysema | 0.05 | 0 | 0.1 | 0 | 0 | 0 |
| Employment | 35.88 | 35.72 | 36 | 5.17 | 7.16 | 3.43 |
| Family health history | 1.23 | 0 | 2.25 | 1.86 | 0.08 | 3.43 |
| Heart failure | 0.18 | 0.4 | 0 | 0 | 0 | 0 |
| Heart valve disease | 0.03 | 0 | 0.05 | 0.14 | 0 | 0.27 |
| Hypertension | 3.22 | 5.12 | 1.66 | 0.49 | 1.04 | 0 |
| IBD | 0 | 0 | 0 | 0 | 0 | 0 |
| Immigration | 0 | 0 | 0 | 1.55 | 3.33 | 0 |
| Inflammation | 2.6 | 1.36 | 3.62 | 0.09 | 0.2 | 0 |
| Kidney function | 1.64 | 0.66 | 2.45 | 0.56 | 0 | 1.05 |
| Life events | 4.26 | 6.28 | 2.6 | 1.59 | 2.41 | 0.87 |
| Living together with others | 0 | 0 | 0 | 0 | 0 | 0 |
| Lung function | 1.04 | 0.93 | 1.13 | 3 | 3.64 | 2.44 |
| MI | 0.05 | 0 | 0.1 | 0.01 | 0.02 | 0 |
| Nutrition | 1.92 | 3.26 | 0.81 | 2.14 | 4.29 | 0.25 |
| OLD | 0.75 | 1.26 | 0.33 | 0.13 | 0 | 0.25 |
| Other lung disease | 0.12 | 0.26 | 0 | 0 | 0 | 0 |
| Other nicotine than cigarettes | 2.25 | 4.49 | 0.4 | 0.07 | 0.16 | 0 |
| Pain | 33.29 | 29.33 | 36.56 | 5.64 | 4.65 | 6.52 |
| Peripheral artery disease | 0 | 0 | 0 | 0.02 | 0 | 0.03 |
| Physical activity | 40.21 | 40.03 | 40.37 | 12.3 | 16.69 | 8.44 |
| Rheumatic disease | 5.32 | 4.34 | 6.13 | 0.46 | 0.34 | 0.57 |
| Sense of control | 26.47 | 28.03 | 25.18 | 18.04 | 17.01 | 18.94 |
| Sleep | 32.65 | 29.71 | 35.08 | 3.64 | 4.8 | 2.62 |
| Smoking | 1.31 | 1.66 | 1.01 | 1.06 | 2.1 | 0.14 |
| Social life | 0 | 0 | 0 | 0.82 | 1.23 | 0.46 |
| Socioeconomic | 4.3 | 7 | 2.06 | 1.84 | 3.07 | 0.77 |
| Stroke | 0 | 0 | 0 | 0 | 0 | 0 |
| Tuberculosis | 0 | 0 | 0 | 0.05 | 0.11 | 0 |
| Women’s health | 1.46 | 0 | 2.66 | 0.61 | 0 | 1.14 |

The factors are the summarised importance scores of similar variables, which were assigned before the analysis. The importance score corresponds to the SHapley Additive exPlanations (SHAP) absolute mean divided by the models’ mean absolute error, which is comparable to the effect size. The SHAP absolute mean corresponds to the average degree of change from the mean score of the Short Form 12 for physical HrQoL (men: 53.4; women: 51.4) by a predictor variable among all participants. Abbreviations: CABG = coronary artery bypass graft; IBD = inflammatory bowel disease; MI = myocardial infarction; OLD = obstructive lung disease; PCI = Percutaneous Coronary Intervention.
