# Supplementary material for: Factors important for health-related quality of life in men and women: The population based SCAPIS study
Source: PLoS One. 2023 Nov 3;18(11):e0294030. doi: 10.1371/journal.pone.0294030 (PMC10624288; doi:10.1371/journal.pone.0294030)
Supplement: S4 Table — The SHAP absolute mean corresponds to the average degree of change from the mean score of the Short Form 12 for physical HrQoL (men: 53.4; women: 51.4) by a predictor variable among all participants. Abbreviations: CABG = coronary artery bypass graft; COPD = Chronic obstructive pulmonary disease; FEV1 = forced expiratory volume in 1 second; FVC = Forced vital capacity; Hb = haemoglobin; HbA1c = haemoglobin A1c; HDL = high-density lipoprotein; hsCRP = High-sensitivity C-reactive protein; IBD = inflammatory bowel disease; LDL = low-density lipoprotein; MEF50 = maximal expiratory flow at 50% of the forced vital capacity; MI = myocardial infarction; OLD = obstructive lung disease; PCI = Percutaneous Coronary Intervention; SLE = Systemic lupus erythematosus; VCmax = Maximum vital capacity. (DOCX) [file pone.0294030.s004.docx]

| **S4 Table - Individual variables’ importance score for physical and mental HrQoL among all participants, men, and women.** | | | | | | | |
| --- | --- | --- | --- | --- | --- | --- | --- |
|  |  | **Physical HRQoL** | | | **Mental HRQoL** | | |
| **Variable** | **Factor** | **All participants** | **Men** | **Women** | **All participants** | **Men** | **Women** |
|  |  |  |  |  |  |  |  |
|  |  |  |  |  |  |  |  |
| Ability to find 20 000 SEK in a week for unforeseen events | Socioeconomic | 0 | 0 | 0 | 0 | 0 | 0 |
| Accelerometer wear time (minutes per day) | Physical activity | 1.06 | 0.94 | 1.15 | 0.93 | 2 | 0 |
| Accelerometer wear time (total minutes) | Physical activity | 0.09 | 0.2 | 0 | 0.23 | 0.49 | 0 |
| Acetylsalicylic acid, last 2 weeks, self-reported | Pain | 0.82 | 0.56 | 1.04 | 0.05 | 0 | 0.09 |
| Action taken at pain or discomfort in chest | Chest pain | 1.96 | 2.78 | 1.29 | 0 | 0 | 0 |
| Age at first menstruation | Women’s health | 0 | 0 | 0 | 0 | 0 | 0 |
| Age at first snus usage occasion | Other nicotine than cigarettes | 0.69 | 1.52 | 0 | 0 | 0 | 0 |
| Age at smoke start (derived variable) | Smoking | 0.68 | 1.5 | 0 | 0.02 | 0.05 | 0 |
| Age at study visit 1, rounded to 1 decimal | Age | 1.1 | 0 | 2.01 | 5.13 | 6 | 4.36 |
| Allergic rhinitis, last 12 months, self-reported | Allergy | 0 | 0 | 0 | 0 | 0 | 0 |
| Angina pectoris, doctor-diagnosed, self-reported | Angina | 0 | 0 | 0 | 0 | 0 | 0 |
| Angina pectoris, medication last 2 weeks, self-reported | Angina | 0 | 0 | 0 | 0 | 0 | 0 |
| Ankle-brachial index | Blood pressure and Pulse | 0.23 | 0.51 | 0 | 0.13 | 0.2 | 0.08 |
| Annoyed someone because of drinking | Alcohol | 0 | 0 | 0 | 0.15 | 0.32 | 0 |
| Aortic intervention, location, self-reported | Aortic intervention | 0 | 0 | 0 | 0 | 0 | 0 |
| Aortic intervention, self-reported | Aortic intervention | 0 | 0 | 0 | 0 | 0 | 0 |
| Aortic intervention, year of intervention, self-reported | Aortic intervention | 0 | 0 | 0 | 0.04 | 0.09 | 0 |
| Approximate weight at 20 years | Body size | 0.69 | 0 | 1.25 | 0.58 | 0.43 | 0.72 |
| Arm of doppler measurement, systolic Blood Pressure. | Blood pressure and Pulse | 0 | 0 | 0 | 0 | 0 | 0 |
| Asthma, age of onset, self-reported | OLD | 0 | 0 | 0 | 0 | 0 | 0 |
| Asthma, doctor-diagnosed, self-reported | OLD | 0 | 0 | 0 | 0 | 0 | 0 |
| Asthma, medication last 2 weeks, self-reported | OLD | 0 | 0 | 0 | 0 | 0 | 0 |
| Atrial fibrillation, doctor-diagnosed, self-reported | Atrial fibrillation | 0 | 0 | 0 | 0 | 0 | 0 |
| Atrial fibrillation, medication last 2 weeks, self-reported | Atrial fibrillation | 0.14 | 0.31 | 0 | 0 | 0 | 0 |
| Attempts to cut down on drinking | Alcohol | 0 | 0 | 0 | 0.57 | 0 | 1.07 |
| Average daily cigarette consumption during years of smoking (derived variable) | Smoking | 0.58 | 0.08 | 0.99 | 0 | 0 | 0 |
| Average income in neighbourhood | Socioeconomic | 1.75 | 1.95 | 1.58 | 0.79 | 1.68 | 0.02 |
| Average physical activity intensity; Mean Vector Magnitude in counts per minute | Physical activity | 2.07 | 3.3 | 1.06 | 0.15 | 0.31 | 0 |
| Bad self-esteem/feeling worthless | Depression | 0 | 0 | 0 | 1.04 | 0 | 1.94 |
| Body height | Body size | 0 | 0 | 0 | 0.31 | 0.66 | 0 |
| Body mass index | Body size | 7.22 | 0.85 | 12.49 | 0.66 | 0.02 | 1.23 |
| Body weight | Body size | 0.2 | 0.22 | 0.18 | 0.94 | 0 | 1.77 |
| Born in Sweden | Immigration | 0 | 0 | 0 | 1.1 | 2.34 | 0 |
| Breathing problems affecting daily activities | Breathlessness | 0.35 | 0.29 | 0.39 | 0 | 0 | 0 |
| Breathless when climbing two flights of stairs | Breathlessness | 0.38 | 0.84 | 0 | 0 | 0 | 0 |
| Breathless when walking on level ground | Breathlessness | 5.07 | 4.17 | 5.81 | 0.68 | 0 | 1.27 |
| Breathlessness preventing leaving home | Breathlessness | 0.31 | 0.16 | 0.43 | 0 | 0 | 0 |
| CABG or PCI intervention, self-reported | CABG or PCI | 0 | 0 | 0 | 0 | 0 | 0 |
| CABG or PCI, type of intervention, self-reported | CABG or PCI | 0 | 0 | 0 | 0 | 0 | 0 |
| CABG or PCI, year of intervention, self-reported | CABG or PCI | 0 | 0 | 0 | 0 | 0 | 0 |
| Calculated nutrition value: alcohol | Alcohol | 1.08 | 1.15 | 1.03 | 0.45 | 0 | 0.85 |
| Calculated nutrition value: carbohydrates | Nutrition | 0 | 0 | 0 | 0.51 | 1.09 | 0 |
| Calculated nutrition value: cholesterol | Nutrition | 0 | 0 | 0 | 0 | 0 | 0 |
| Calculated nutrition value: energy (kcal) | Nutrition | 0.04 | 0.09 | 0 | 0.28 | 0.61 | 0 |
| Calculated nutrition value: fat | Nutrition | 1.23 | 1.75 | 0.81 | 0.08 | 0.17 | 0 |
| Calculated nutrition value: protein | Nutrition | 0.64 | 1.42 | 0 | 1.27 | 2.42 | 0.25 |
| Cancer, doctor-diagnosed, self-reported | Cancer | 0 | 0 | 0 | 0 | 0 | 0 |
| Cancer, medication last 2 weeks, self-reported | Cancer | 0 | 0 | 0 | 0 | 0 | 0 |
| Cancer, year of diagnosis (year - yyyy) | Cancer | 0.03 | 0.07 | 0 | 0 | 0 | 0 |
| Cardiovascular risk SCORE-value | Cardiovascular risk | 0 | 0 | 0 | 0.68 | 0 | 1.28 |
| Celiac disease, doctor-diagnosed, self-reported | Celiac disease | 0 | 0 | 0 | 0 | 0 | 0 |
| Celiac disease, year of diagnosis, self-reported | Celiac disease | 0.11 | 0.24 | 0 | 0.02 | 0.04 | 0 |
| Chance of dozing: as a passenger in a car for an hour without a break | Sleep | 0.53 | 0 | 0.97 | 0.24 | 0 | 0.46 |
| Chance of dozing: in a car, while stopped for a few minutes in the traffic | Sleep | 0.19 | 0 | 0.35 | 0.7 | 0 | 1.32 |
| Chance of dozing: lying down to rest in the afternoon when circumstances permit | Sleep | 2.93 | 1.08 | 4.46 | 1.02 | 2.19 | 0 |
| Chance of dozing: sitting and reading | Sleep | 0 | 0 | 0 | 0 | 0 | 0 |
| Chance of dozing: sitting and talking to someone | Sleep | 0 | 0 | 0 | 1.11 | 2.38 | 0 |
| Chance of dozing: sitting inactive in a public place (e.g. a theatre or a meeting) | Sleep | 0.88 | 0 | 1.61 | 0 | 0 | 0 |
| Chance of dozing: sitting quietly after a lunch without alcohol | Sleep | 0.23 | 0.5 | 0 | 0 | 0 | 0 |
| Chance of dozing: watching TV | Sleep | 0 | 0 | 0 | 0 | 0 | 0 |
| Cholesterol Numerical result | Cholesterol | 0.27 | 0.59 | 0 | 0 | 0 | 0 |
| Chronic bronchitis, doctor-diagnosed, self-reported | Chronic bronchitis | 0 | 0 | 0 | 0 | 0 | 0 |
| Chronic rhinosinusitis (CRS), doctor-diagnosed, self-reported | Allergy | 0 | 0 | 0 | 0 | 0 | 0 |
| Classification of chronic airway limitation based on value of FEV1/FVC | Lung function | 0 | 0 | 0 | 0 | 0 | 0 |
| Classification of glycaemic status | Diabetes | 0 | 0 | 0 | 0 | 0 | 0 |
| Cohabitants smoking at home | Smoking | 0 | 0 | 0 | 0 | 0 | 0 |
| Concentration problems | Depression | 0 | 0 | 0 | 4.75 | 5.48 | 4.11 |
| Concerns expressed by others about drinking | Alcohol | 0 | 0 | 0 | 0.28 | 0.6 | 0 |
| COPD, chronic bronchitis or emphysema, Doctor-diagnosed, self-reported | OLD | 0.18 | 0 | 0.33 | 0 | 0 | 0 |
| COPD, chronic bronchitis or emphysema, medication last 2 weeks, doctor-diagnosed, self-reported | OLD | 0 | 0 | 0 | 0 | 0 | 0 |
| COPD, doctor-diagnosed, self-reported | OLD | 0 | 0 | 0 | 0 | 0 | 0 |
| Coronary dominance | Blood pressure and Pulse | 0 | 0 | 0 | 0 | 0 | 0 |
| Coughing most days for at least three months every year | Coughing | 0 | 0 | 0 | 0 | 0 | 0 |
| Coughing when not having a cold | Coughing | 3.07 | 4.99 | 1.48 | 0 | 0 | 0 |
| Creatinine Numerical result | Kidney function | 1.64 | 0.66 | 2.45 | 0.56 | 0 | 1.05 |
| Crohn's disease or ulcerative colitis, doctor-diagnosed, self-reported | IBD | 0 | 0 | 0 | 0 | 0 | 0 |
| Crohn's disease or ulcerative colitis, medication, self-reported | IBD | 0 | 0 | 0 | 0 | 0 | 0 |
| Crohn's disease or ulcerative colitis, year of diagnosis, self-reported | IBD | 0 | 0 | 0 | 0 | 0 | 0 |
| Current smoking status, self-reported | Smoking | 0 | 0 | 0 | 0 | 0 | 0 |
| Current snus users: number of snus cans per week | Other nicotine products than cigarettes | 0.22 | 0 | 0.4 | 0 | 0 | 0 |
| Daily usage of other nicotine products | Other nicotine products than cigarettes | 0 | 0 | 0 | 0.07 | 0.16 | 0 |
| Daily usage of snus for more than one month | Other nicotine products than cigarettes | 0 | 0 | 0 | 0 | 0 | 0 |
| Daily use of chewing tobacco | Other nicotine products than cigarettes | 0 | 0 | 0 | 0 | 0 | 0 |
| Daily use of nicotine replacements | Other nicotine products than cigarettes | 0 | 0 | 0 | 0 | 0 | 0 |
| Degree of physical acitivity at work | Physical activity | 4.89 | 8.34 | 2.03 | 0.8 | 0.5 | 1.06 |
| Diabetes, age of onset, self-reported | Diabetes | 0.94 | 1.5 | 0.47 | 0.02 | 0 | 0.05 |
| Diabetes, doctor-diagnosed, self-reported | Diabetes | 0 | 0 | 0 | 0 | 0 | 0 |
| Diabetes, medication last 2 weeks, self-reported | Diabetes | 0 | 0 | 0 | 0 | 0 | 0 |
| Diastolic blood pressure, mean brachial (recommended for general analysis) | Blood pressure and Pulse | 0 | 0 | 0 | 0 | 0 | 0 |
| Diclofenac doses last 2 weeks | Pain | 5.8 | 6.85 | 4.93 | 2.16 | 2.73 | 1.67 |
| Difficulties managing regular expenses, last 12 months | Socioeconomic | 0 | 0 | 0 | 0.07 | 0 | 0.14 |
| Diffusion capacity for carbon monoxide | Lung function | 0.48 | 0.93 | 0.11 | 0.05 | 0 | 0.1 |
| Diffusion capacity for carbon monoxide, related to alveolar volume | Lung function | 0 | 0 | 0 | 0.83 | 1.18 | 0.52 |
| Discoloured nasal discharge, >12 weeks during last 12 months, self-reported | Allergy | 1.05 | 1.73 | 0.49 | 0 | 0 | 0 |
| Divorced | Living together with others | 0 | 0 | 0 | 0 | 0 | 0 |
| Drinks alcoholic cider and soda | Alcohol | 0 | 0 | 0 | 0 | 0 | 0 |
| Drinks beer | Alcohol | 0 | 0 | 0 | 0 | 0 | 0 |
| Drinks hard liquor (incl. drinks, whiskey, liqueur etc.) | Alcohol | 0 | 0 | 0 | 0 | 0 | 0 |
| Drinks wine | Alcohol | 1.23 | 0 | 2.25 | 0 | 0 | 0 |
| Early retirement pension or sickness pension | Employment | 9.66 | 6.06 | 12.63 | 0 | 0 | 0 |
| Emphysema, doctor-diagnosed, self-reported | Emphysema | 0.05 | 0 | 0.1 | 0 | 0 | 0 |
| Ever had severe pain in chest >30 min | Chest pain | 0.19 | 0 | 0.34 | 0 | 0 | 0 |
| Ex-smokers: average grams of pipe tobacco per day – ex-smoker | Smoking | 0 | 0 | 0 | 0 | 0 | 0 |
| Ex-smokers: average number of cigarettes per day | Smoking | 0 | 0 | 0 | 0 | 0 | 0 |
| Ex-smokers: average number of cigars/cigar-cigarettes per day | Smoking | 0 | 0 | 0 | 0 | 0 | 0 |
| Expectance of mostly positive experiences next 5-10 years | Sense of control | 3.66 | 6.85 | 1.02 | 0.56 | 0 | 1.05 |
| Experience of stress | Sense of control | 1.41 | 1.12 | 1.66 | 13.68 | 11.18 | 15.87 |
| Extent of gainful employment (% of fulltime) | Employment | 10.63 | 7.32 | 13.36 | 0.61 | 0.42 | 0.77 |
| Facial pain or pressure, >12 weeks during last 12 months, self-reported | Allergy | 3.32 | 3.24 | 3.38 | 0 | 0 | 0 |
| Family history of asthma, any first degree relative | Family health history | 0.7 | 0 | 1.28 | 0 | 0 | 0 |
| Family history of asthma, subject's children | Family health history | 0 | 0 | 0 | 0 | 0 | 0 |
| Family history of asthma, subject's father | Family health history | 0 | 0 | 0 | 0 | 0 | 0 |
| Family history of asthma, subject's mother | Family health history | 0 | 0 | 0 | 0 | 0 | 0 |
| Family history of asthma, subject's sibling | Family health history | 0 | 0 | 0 | 0 | 0 | 0 |
| Family history of bronchitis, COPD or emphysema, any first degree relative | Family health history | 0.53 | 0 | 0.98 | 0.55 | 0 | 1.04 |
| Family history of bronchitis, COPD or emphysema, subject´s children | Family health history | 0 | 0 | 0 | 0 | 0 | 0 |
| Family history of bronchitis, COPD or emphysema, subject´s father | Family health history | 0 | 0 | 0 | 0 | 0 | 0 |
| Family history of bronchitis, COPD or emphysema, subject´s mother | Family health history | 0 | 0 | 0 | 0 | 0 | 0 |
| Family history of bronchitis, COPD or emphysema, subject´s siblings | Family health history | 0 | 0 | 0 | 0.17 | 0 | 0.31 |
| Family history of diabetes, any first degree relative | Family health history | 0 | 0 | 0 | 0 | 0 | 0 |
| Family history of diabetes, subject's children | Family health history | 0 | 0 | 0 | 0 | 0 | 0 |
| Family history of diabetes, subject's father | Family health history | 0 | 0 | 0 | 0.38 | 0 | 0.71 |
| Family history of diabetes, subject's mother | Family health history | 0 | 0 | 0 | 0 | 0 | 0 |
| Family history of diabetes, subject's sibling | Family health history | 0 | 0 | 0 | 0 | 0 | 0 |
| Family history of lung cancer, subject's father | Family health history | 0 | 0 | 0 | 0 | 0 | 0 |
| Family history of lung cancer, subject's mother | Family health history | 0 | 0 | 0 | 0 | 0 | 0 |
| Family history of lung cancer, subject´s parent or sibling | Family health history | 0 | 0 | 0 | 0 | 0 | 0 |
| Family history of lung cancer, subject's sibling | Family health history | 0 | 0 | 0 | 0 | 0 | 0 |
| Family history of myocardial infarction, subject's father | Family health history | 0 | 0 | 0 | 0 | 0 | 0 |
| Family history of myocardial infarction, subject's mother | Family health history | 0 | 0 | 0 | 0 | 0 | 0 |
| Family history of myocardial infarction, subject´s parent or sibling | Family health history | 0 | 0 | 0 | 0 | 0 | 0 |
| Family history of myocardial infarction, subject's sibling | Family health history | 0 | 0 | 0 | 0 | 0 | 0 |
| Family history of stroke, subject's father | Family health history | 0 | 0 | 0 | 0 | 0 | 0 |
| Family history of stroke, subject's mother | Family health history | 0 | 0 | 0 | 0 | 0 | 0 |
| Family history of stroke, subject´s parent or sibling | Family health history | 0 | 0 | 0 | 0 | 0 | 0 |
| Family history of stroke, subject's sibling | Family health history | 0 | 0 | 0 | 0.31 | 0.08 | 0.51 |
| Father's biological figure at 40 years of age | Family health history | 0 | 0 | 0 | 0.46 | 0 | 0.86 |
| Feeling of being unfairly treated | Sense of control | 3.56 | 4.94 | 2.42 | 0.56 | 0 | 1.05 |
| Feelings of sadness/depression, last 12 months | Depression | 0 | 0 | 0 | 0.46 | 0 | 0.86 |
| Felt guilt about drinking | Alcohol | 0 | 0 | 0 | 0.44 | 0.93 | 0 |
| Felt tired or low on energy | Depression | 4.1 | 0 | 7.5 | 1.08 | 1.13 | 1.03 |
| FEV1/FVC post bronchodilatation | Lung function | 0.56 | 0 | 1.02 | 0 | 0 | 0 |
| FEV1/VCmax post bronchodilatation | Lung function | 0 | 0 | 0 | 0.13 | 0.29 | 0 |
| Forced expiratory volume in one second post-bronchodilation | Lung function | 0 | 0 | 0 | 0.01 | 0.02 | 0 |
| Forced vital capacity (FVC) post bronchodilation. | Lung function | 0 | 0 | 0 | 1.41 | 2.15 | 0.76 |
| Former snus user: number of snus cans per week | Other nicotine products than cigarettes | 0 | 0 | 0 | 0 | 0 | 0 |
| Frequency drinking resulting in blackouts, last year | Alcohol | 0 | 0 | 0 | 0 | 0 | 0 |
| Frequency failing to do what is expected due to drinking, last year | Alcohol | 0 | 0 | 0 | 0.37 | 0 | 0.69 |
| Frequency feeling guilty about drinking, last year | Alcohol | 0 | 0 | 0 | 0.12 | 0.25 | 0 |
| Frequency having >6 alcoholic drinks on one occasion | Alcohol | 0.67 | 0 | 1.23 | 0 | 0 | 0 |
| Frequency having an alcoholic drink, last year | Alcohol | 0.57 | 1.27 | 0 | 0 | 0 | 0 |
| Frequency needing a drink in the morning after drinking the night before, last year | Alcohol | 0 | 0 | 0 | 0.11 | 0.07 | 0.14 |
| Frequency not being able to stop drinking, last year | Alcohol | 0 | 0 | 0 | 0 | 0 | 0 |
| Frequency of diffuculty to fall asleep at night | Sleep | 1.82 | 0 | 3.32 | 0.04 | 0.08 | 0 |
| Frequency of loud snoring (according to self or others) | Sleep | 0.43 | 0 | 0.78 | 0 | 0 | 0 |
| Frequency of reflux after going to bed | Sleep | 2.99 | 3.02 | 2.96 | 0 | 0 | 0 |
| Frequency of waking up several times during the night | Sleep | 4.51 | 4.87 | 4.21 | 0.45 | 0 | 0.84 |
| Frequency of waking up too early without being able to go back to sleep | Sleep | 0 | 0 | 0 | 0.07 | 0.15 | 0 |
| Gained or lost weight | Depression | 1.87 | 4.12 | 0 | 0 | 0 | 0 |
| Gestational diabetes, self-reported | Women’s health | 0 | 0 | 0 | 0 | 0 | 0 |
| Given up trying to improve life | Sense of control | 5.99 | 8.27 | 4.1 | 0.86 | 0.75 | 0.96 |
| Glucose, capillary, day 1 | Diabetes | 0 | 0 | 0 | 0.04 | 0.09 | 0 |
| Glucose, venous, day 1, numerical result | Diabetes | 0 | 0 | 0 | 0 | 0 | 0 |
| Hb Numerical Result | Anaemia | 0.27 | 0.52 | 0.06 | 0.68 | 1.02 | 0.37 |
| HbA1c Numerical result | Diabetes | 0 | 0 | 0 | 0.43 | 0.87 | 0.06 |
| HDL Numerical result - national variable. | Cholesterol | 0.01 | 0 | 0.02 | 0 | 0 | 0 |
| Heart failure, medication last 2 weeks, self-reported | Heart failure | 0.18 | 0.4 | 0 | 0 | 0 | 0 |
| Heart failure, self-reported, doctor-diagnosed | Heart failure | 0 | 0 | 0 | 0 | 0 | 0 |
| Heart valve disease, doctor-diagnosed, self-reported | Heart valve disease | 0 | 0 | 0 | 0 | 0 | 0 |
| Heart valve disease, type, self-reported | Heart valve disease | 0.03 | 0 | 0.05 | 0.14 | 0 | 0.27 |
| Heart valve disease, year of intervention, self-reported | Heart valve disease | 0 | 0 | 0 | 0 | 0 | 0 |
| High degree of unexpected changes in life, past 10 years | Sense of control | 3.96 | 3.24 | 4.56 | 0.98 | 2.11 | 0 |
| Highest completed level of education | Education | 0 | 0 | 0 | 0 | 0 | 0 |
| Hip circumference | Body size | 1.16 | 1.14 | 1.18 | 0.21 | 0.46 | 0 |
| Hormone treatment of menopausal symptoms, self-reported | Women’s health | 1.46 | 0 | 2.66 | 0.44 | 0 | 0.82 |
| Hours bicycling: winter | Physical activity | 0 | 0 | 0 | 0.11 | 0.24 | 0 |
| Hours of sleep per night under usual circumstances | Sleep | 1.24 | 0 | 2.26 | 0 | 0 | 0 |
| Hours spent bicycling weekly in the summer | Physical activity | 0.57 | 0 | 1.05 | 0.32 | 0.53 | 0.13 |
| Hours spent walking weekly in the summer | Physical activity | 0 | 0 | 0 | 1.47 | 3.14 | 0 |
| Hours spent walking weekly in the winter | Physical activity | 0.22 | 0 | 0.39 | 0.6 | 0.07 | 1.06 |
| hsCRP Numerical result - national variable | Inflammation | 2.6 | 1.36 | 3.62 | 0.09 | 0.2 | 0 |
| Hyperlipidaemia, doctor-diagnosed, self-reported | Cholesterol | 0 | 0 | 0 | 0 | 0 | 0 |
| Hyperlipidaemia, medication last 2 weeks, self-reported | Cholesterol | 0 | 0 | 0 | 0 | 0 | 0 |
| Hypertension, doctor-diagnosed, self-reported | Hypertension | 2.32 | 5.12 | 0 | 0 | 0 | 0 |
| Hypertension, medication last 2 weeks, self-reported | Hypertension | 0.91 | 0 | 1.66 | 0.49 | 1.04 | 0 |
| If you did not have a period, what was the reason? Response alternative: medication. | Women’s health | 0 | 0 | 0 | 0.17 | 0 | 0.32 |
| Injuries caused by drinking | Alcohol | 0 | 0 | 0 | 0.16 | 0.34 | 0 |
| LDL Numerical result - national variable. | Cholesterol | 0.24 | 0.54 | 0 | 0.58 | 1.02 | 0.19 |
| Life events: concerns for someone close | Life events | 1.17 | 2.58 | 0 | 0 | 0 | 0 |
| Life events: death of someone close | Life events | 0 | 0 | 0 | 0.53 | 1.13 | 0 |
| Life events: felt insecure at work | Life events | 0 | 0 | 0 | 0.33 | 0.7 | 0 |
| Life events: had to change housing | Life events | 0 | 0 | 0 | 0 | 0 | 0 |
| Life events: had to change job | Life events | 0 | 0 | 0 | 0.46 | 0 | 0.87 |
| Life events: loss of job | Life events | 0 | 0 | 0 | 0 | 0 | 0 |
| Life events: own divorce of separation | Life events | 0.26 | 0.58 | 0 | 0 | 0 | 0 |
| Life events: received criminal penalty | Life events | 0 | 0 | 0 | 0.16 | 0.34 | 0 |
| Life events: serious financial problems | Life events | 0.91 | 0 | 1.66 | 0.12 | 0.25 | 0 |
| Life events: serious illness/accident in family | Life events | 1.93 | 3.12 | 0.94 | 0 | 0 | 0 |
| Living Alone | Living together with others | 0 | 0 | 0 | 0 | 0 | 0 |
| Living in own apartment | Socioeconomic | 0 | 0 | 0 | 0 | 0 | 0 |
| Living in rental apartment | Socioeconomic | 0 | 0 | 0 | 0 | 0 | 0 |
| Living in villa | Socioeconomic | 0 | 0 | 0 | 0 | 0 | 0 |
| Sense of control: at work | Sense of control | 0 | 0 | 0 | 0.81 | 1.74 | 0 |
| Lost interest in things that usually gives pleasure | Depression | 0 | 0 | 0 | 4.71 | 3.99 | 5.34 |
| Low-intensity physical activity (LIPA), average minutes per day | Physical activity | 0 | 0 | 0 | 0 | 0 | 0 |
| Low-intensity physical activity (LIPA), percentage of wear time | Physical activity | 0 | 0 | 0 | 0 | 0 | 0 |
| Low-intensity physical activity (LIPA), total minutes | Physical activity | 0.18 | 0.4 | 0 | 0.12 | 0.27 | 0 |
| Lung disease (other than COPD, chronic bronchitis or emphysema), doctor diagnosed, self-reported | Other lung disease | 0.12 | 0.26 | 0 | 0 | 0 | 0 |
| Married | Living together with others | 0 | 0 | 0 | 0 | 0 | 0 |
| MEF50 post bronchodilatation | Lung function | 0 | 0 | 0 | 0.57 | 0 | 1.07 |
| Menstruation, last year | Women’s health | 0 | 0 | 0 | 0 | 0 | 0 |
| Minutes spent sitting, last 7 days | Physical activity | 1.31 | 0 | 2.39 | 2.26 | 4.84 | 0 |
| Mode of travel to work: autumn | Physical activity | 0 | 0 | 0 | 0 | 0 | 0 |
| Mode of travel to work: spring | Physical activity | 1.46 | 0 | 2.66 | 0 | 0 | 0 |
| Mode of travel to work: summer | Physical activity | 0 | 0 | 0 | 0 | 0 | 0 |
| Mode of travel to work: winter | Physical activity | 0.72 | 0 | 1.32 | 0 | 0 | 0 |
| Moderate- and vigorous-intensity physical activity (MVPA), average minutes per day | Physical activity | 0.9 | 1.5 | 0.41 | 0.47 | 0 | 0.89 |
| Moderate- and vigorous-intensity physical activity (MVPA), percentage of wear time | Physical activity | 0 | 0 | 0 | 0.16 | 0 | 0.29 |
| Moderate- and vigorous-intensity physical activity (MVPA), total minutes | Physical activity | 2.83 | 0 | 5.16 | 0.75 | 0 | 1.41 |
| Moderate-intensity physical activity (MPA), average minutes per day | Physical activity | 0.99 | 0 | 1.8 | 0 | 0 | 0 |
| Moderate-intensity physical activity (MPA), percentage of wear time | Physical activity | 0 | 0 | 0 | 0 | 0 | 0 |
| Moderate intensity physical activity (MPA), total minutes | Physical activity | 0 | 0 | 0 | 0.02 | 0 | 0.04 |
| Month of accelerometer recording | Physical activity | 0 | 0 | 0 | 0 | 0 | 0 |
| Mother's biological figure at 40 years of age | Family health history | 0 | 0 | 0 | 0 | 0 | 0 |
| Myocardial infarction, doctor-diagnosed, self-reported | MI | 0 | 0 | 0 | 0 | 0 | 0 |
| Myocardial infarction, year of first event, self-reported | MI | 0 | 0 | 0 | 0 | 0 | 0 |
| Myocardial infarction, year of latest event, self-reported | MI | 0.05 | 0 | 0.1 | 0.01 | 0.02 | 0 |
| Nasal Obstruction, >12 weeks during last 12 months, self-reported | Allergy | 1.58 | 0 | 2.89 | 0.33 | 0 | 0.62 |
| Need to stop for breath after 100 meters | Breathlessness | 0.15 | 0.34 | 0 | 0 | 0 | 0 |
| Need to stop for breath when walking on level ground | Breathlessness | 0.35 | 0.78 | 0 | 0.09 | 0.2 | 0 |
| Non-steroidal anti-inflammatory drugs (NSAID) doses last 2 weeks | Pain | 3.27 | 3.74 | 2.89 | 0 | 0 | 0 |
| Not enough time to complete tasks at work | Employment | 2.78 | 3.95 | 1.81 | 2.25 | 4.82 | 0 |
| Not feeling in control of life | Sense of control | 7.89 | 3.61 | 11.42 | 0.58 | 1.24 | 0 |
| Number of alcoholic cider bottle/cans (>4.5 vol% alcohol) consumed during an average month | Alcohol | 0 | 0 | 0 | 0 | 0 | 0 |
| Number of alcoholic cider/soda bottles/cans consumed during an average month | Alcohol | 0.25 | 0.55 | 0 | 0 | 0 | 0 |
| Number of alcoholic drinks on a typical drinking day | Alcohol | 0 | 0 | 0 | 0 | 0 | 0 |
| Number of days hospitalized due to breathing problems, last 12 months | Breathlessness | 0 | 0 | 0 | 0 | 0 | 0 |
| Number of episodes with breathing problems requiring health care, last 12 months | Breathlessness | 0 | 0 | 0 | 0 | 0 | 0 |
| Number of episodes with breathing problems requiring hospitalization, last 12 months | Breathlessness | 0 | 0 | 0 | 0 | 0 | 0 |
| Number of episodes with breathing problems, last 12 months | Breathlessness | 0 | 0 | 0 | 0 | 0 | 0 |
| Number of extra strong beer bottles/cans (approx >5.2 vol% alcohol) consumed during an average month | Alcohol | 0 | 0 | 0 | 0.1 | 0.22 | 0 |
| Number of medium-strong beer bottles/cans (2.26-3.5 vol% alcohol) consumed during an average month | Alcohol | 0 | 0 | 0 | 0 | 0 | 0 |
| Number of medium-strong beer bottles/cans (approx 3.5-4.5 vol% alcohol) consumed during an average month | Alcohol | 0 | 0 | 0 | 0 | 0 | 0 |
| Number of occasions of physical exercise, last 3 months | Physical activity | 3.05 | 4.82 | 1.6 | 0.65 | 1.07 | 0.28 |
| Number of paracetamol doses last 2 weeks | Pain | 23.39 | 18.17 | 27.7 | 3.43 | 1.92 | 4.75 |
| Number of strong beer bottles/cans (approx 4.5-5.2 vol% alcohol) consumed during an average month | Alcohol | 0 | 0 | 0 | 0.62 | 1.33 | 0 |
| Number of valid days of accelerometer recording | Physical activity | 0 | 0 | 0 | 0.58 | 1.24 | 0 |
| Number of weekend days of accelerometer recording | Physical activity | 0 | 0 | 0 | 0 | 0 | 0 |
| Number of wine bottles consumed during an average month | Alcohol | 0 | 0 | 0 | 0 | 0 | 0 |
| Number of years of smoking (derived variable) | Smoking | 0 | 0 | 0 | 0.03 | 0.07 | 0 |
| Other type of living | Socioeconomic | 0 | 0 | 0 | 0 | 0 | 0 |
| Pack years for cigarettes (derived variable) | Smoking | 0 | 0 | 0 | 0.17 | 0.19 | 0.14 |
| Pain in calves when hurrying or walking uphill | Claudication | 0 | 0 | 0 | 0.07 | 0.15 | 0 |
| Pain or discomfort in chest when hurrying or walking uphill | Chest pain | 0 | 0 | 0 | 0 | 0 | 0 |
| Pain or discomfort in chest when walking on level ground | Chest pain | 0 | 0 | 0 | 0 | 0 | 0 |
| Parental heredity for myocardial infarction | Family health history | 0 | 0 | 0 | 0 | 0 | 0 |
| Parental heredity for stroke | Family health history | 0 | 0 | 0 | 0 | 0 | 0 |
| Percentage of weekend days of accelerometer recording | Physical activity | 0 | 0 | 0 | 0 | 0 | 0 |
| Peripheral artery disease, intervention, self-reported | Peripheral artery disease | 0 | 0 | 0 | 0 | 0 | 0 |
| Peripheral artery disease, type of intervention, self-reported | Peripheral artery disease | 0 | 0 | 0 | 0 | 0 | 0 |
| Peripheral artery disease, year of intervention, self-reported | Peripheral artery disease | 0 | 0 | 0 | 0.02 | 0 | 0.03 |
| Phlegm problems >3 months per year | Coughing | 0 | 0 | 0 | 0 | 0 | 0 |
| Phlegm problems when not having a cold | Coughing | 0.44 | 0.96 | 0 | 0 | 0 | 0 |
| Physical activity during leisure time, last 12 months | Physical activity | 11.57 | 13.06 | 10.34 | 0.78 | 1.66 | 0 |
| Polycystic ovary syndrome, self-reported | Women’s health | 0 | 0 | 0 | 0 | 0 | 0 |
| Percentage unemployed in neighbourhood | Socioeconomic | 0 | 0 | 0 | 0 | 0 | 0 |
| Percentage with financial aid in neighbourhood | Socioeconomic | 0 | 0 | 0 | 0 | 0 | 0 |
| Percentage with foreign background in neighbourhood | Socioeconomic | 1.53 | 2.8 | 0.48 | 0.91 | 1.26 | 0.61 |
| Percentage with low economical status in neighbourhood | Socioeconomic | 0 | 0 | 0 | 0 | 0 | 0 |
| Percentage with university degree in neighbourhood | Socioeconomic | 1.02 | 2.25 | 0 | 0.06 | 0.13 | 0 |
| Presence of any carotid artery plaque | Stroke | 0 | 0 | 0 | 0 | 0 | 0 |
| Present occupation: Contractual pension | Employment | 0.42 | 0.49 | 0.35 | 0 | 0 | 0 |
| Present occupation: Gainfully employed | Employment | 0 | 0 | 0 | 0 | 0 | 0 |
| Present occupation: Labour market measures | Employment | 0 | 0 | 0 | 0 | 0 | 0 |
| Present occupation: Leave of absence or parental leave | Employment | 0 | 0 | 0 | 0 | 0 | 0 |
| Present occupation: Long term sick listed (more than 3 months) | Employment | 5.15 | 4.17 | 5.96 | 0 | 0 | 0 |
| Present occupation: Old age pensioner | Employment | 0 | 0 | 0 | 0 | 0 | 0 |
| Present occupation: Other - not gainfully employed | Employment | 0 | 0 | 0 | 0 | 0 | 0 |
| Present occupation: Studying or training | Employment | 0 | 0 | 0 | 0 | 0 | 0 |
| Present occupation: Unemployed | Employment | 0 | 0 | 0 | 0 | 0 | 0 |
| Professional work, last 12 months | Employment | 0 | 0 | 0 | 0 | 0 | 0 |
| Prolonged sedentary (prolonged SED), average minutes per day | Physical activity | 0.06 | 0 | 0.11 | 0 | 0 | 0 |
| Prolonged sedentary (prolonged SED), total minutes | Physical activity | 0 | 0 | 0 | 0.38 | 0.15 | 0.59 |
| Pulse rate | Blood pressure and Pulse | 0.06 | 0.13 | 0 | 1.46 | 0.03 | 2.7 |
| Quality of sleep under usual circumstances | Sleep | 16.91 | 20.24 | 14.16 | 0 | 0 | 0 |
| Quincke edema, self-reported | Allergy | 0.57 | 0.41 | 0.69 | 0 | 0 | 0 |
| Reason for not having menstruation: menopause | Women’s health | 0 | 0 | 0 | 0 | 0 | 0 |
| Reduced sense of smell, >12 weeks during last 12 months, self-reported | Allergy | 0 | 0 | 0 | 0 | 0 | 0 |
| Regular/occasional smokers: average grams of pipe tobacco per day during years of smoking | Smoking | 0.04 | 0.08 | 0 | 0.04 | 0.09 | 0 |
| Regular/occasional smokers: average number of cigarettes per day during years of smoking | Smoking | 0 | 0 | 0 | 0 | 0 | 0 |
| Regular/occasional smokers: average number of cigars/cigarr-cigarettes per day during years of smoking | Smoking | 0.01 | 0 | 0.02 | 0 | 0 | 0 |
| Regular/occasional smokers: grams of pipe tobacco per day presently | Smoking | 0 | 0 | 0 | 0.45 | 0.97 | 0 |
| Regular/occasional smokers: number of cigarettes per day presently | Smoking | 0 | 0 | 0 | 0.34 | 0.74 | 0 |
| Regular/occasional smokers: number of cigars/cigar-cigarettes per day presently | Smoking | 0 | 0 | 0 | 0 | 0 | 0 |
| Rheumatic disease (e.g. rheumatoid arthritis, Bechterew's disease, psoriatic arthritis, SLE, Sjögren's syndrome), doctor-diagnosed, self-reported | Rheumatic disease | 4.15 | 3.56 | 4.63 | 0 | 0 | 0 |
| Rheumatic disease (e.g. rheumatoid arthritis, Bechterews disease, psoriatic arthritis, SLE, Sjögren's syndrome), medication last 2 weeks, self-reported | Rheumatic disease | 0.91 | 0.78 | 1.01 | 0.46 | 0.34 | 0.57 |
| Rheumatic disease (e.g. rheumatoid arthritis, Bechterews disease, psoriatic arthritis, SLE, Sjögren's syndrome), year of diagnosis, self-reported | Rheumatic disease | 0.26 | 0 | 0.48 | 0 | 0 | 0 |
| Sedentary (SED), average minutes per day | Physical activity | 0.27 | 0 | 0.49 | 0 | 0 | 0 |
| Sedentary (SED), percentage of wear time | Physical activity | 0 | 0 | 0 | 0.06 | 0.12 | 0 |
| Sedentary (SED), total minutes | Physical activity | 0.35 | 0 | 0.64 | 0 | 0 | 0 |
| Seep Apnoea, doctor-diagnosed, self-reported | Apnoea | 0.52 | 1.15 | 0 | 0 | 0 | 0 |
| Sharing household with children | Living together with others | 0 | 0 | 0 | 0 | 0 | 0 |
| Sharing household with no one | Living together with others | 0 | 0 | 0 | 0 | 0 | 0 |
| Sharing household with other adults | Living together with others | 0 | 0 | 0 | 0 | 0 | 0 |
| Sharing household with parents/siblings | Living together with others | 0 | 0 | 0 | 0 | 0 | 0 |
| Sharing household with spouse/partner | Living together with others | 0 | 0 | 0 | 0 | 0 | 0 |
| Short of breath when hurrying | Breathlessness | 11.13 | 9.81 | 12.21 | 0 | 0 | 0 |
| Sleep apnoea or breathing problems during sleep (according to self or others) | Apnoea | 2.75 | 0 | 5.02 | 0 | 0 | 0 |
| Sleep apnoea, year of diagnosis, self-reported | Apnoea | 0 | 0 | 0 | 0.18 | 0.38 | 0 |
| Slow vital capacity (SVC) post bronchodilation. | Lung function | 0 | 0 | 0 | 0 | 0 | 0 |
| Social attachment: existence of person for comfort | Social life | 0 | 0 | 0 | 0 | 0 | 0 |
| Social attachment: existence of person to share feelings of happiness | Social life | 0 | 0 | 0 | 0 | 0 | 0 |
| Social interaction: number of people in household | Social life | 0 | 0 | 0 | 0 | 0 | 0 |
| Social interaction: number of people met during an ordinary week | Social life | 0 | 0 | 0 | 0.24 | 0 | 0.46 |
| Social interaction: number of people who can be easily asked for assistance | Social life | 0 | 0 | 0 | 0 | 0 | 0 |
| Social interaction: number of people who can be turned to in difficulties | Social life | 0 | 0 | 0 | 0.57 | 1.23 | 0 |
| Started to work in current profession (year) | Employment | 0 | 0 | 0 | 0 | 0 | 0 |
| Stroke, doctor-diagnosed, self-reported | Stroke | 0 | 0 | 0 | 0 | 0 | 0 |
| Stroke, year of first event, self-reported | Stroke | 0 | 0 | 0 | 0 | 0 | 0 |
| Stroke, year of latest event, self-reported | Stroke | 0 | 0 | 0 | 0 | 0 | 0 |
| Systolic blood pressure | Blood pressure and Pulse | 0.47 | 0.99 | 0.05 | 0.06 | 0.1 | 0.03 |
| Taking a drink in the morning after a drinking session | Alcohol | 0.4 | 0.89 | 0 | 0 | 0 | 0 |
| TG Numerical result - national variable. | Cholesterol | 0 | 0 | 0 | 0.27 | 0.58 | 0 |
| Thoughts about death | Depression | 0 | 0 | 0 | 0 | 0 | 0 |
| Time for pain or discomfort in chest to disappear | Chest pain | 0 | 0 | 0 | 0 | 0 | 0 |
| Travel distance to work (km) | Physical activity | 0.74 | 0.42 | 1 | 1.46 | 0.06 | 2.69 |
| Trouble falling asleep | Depression | 0.49 | 0 | 0.89 | 0 | 0 | 0 |
| Tuberculosis, doctor-diagnosed, self-reported | Tuberculosis | 0 | 0 | 0 | 0 | 0 | 0 |
| Tuberculosis, year of diagnosis, self-reported | Tuberculosis | 0 | 0 | 0 | 0.05 | 0.11 | 0 |
| Urticaria, self-reported | Allergy | 3.75 | 3.21 | 4.19 | 0 | 0 | 0 |
| Vigorous-intensity physical activity, average minutes per day | Physical activity | 0 | 0 | 0 | 0 | 0 | 0 |
| Vigorous-intensity physical activity, percentage of wear time | Physical activity | 0 | 0 | 0 | 0 | 0 | 0 |
| Vigorous-intensity physical activity, total minutes | Physical activity | 6.89 | 7.06 | 6.75 | 0 | 0 | 0 |
| Volume of hard liquor in a usual drink | Alcohol | 0 | 0 | 0 | 0 | 0 | 0 |
| Waist-Hip Ratio | Body size | 0.04 | 0 | 0.07 | 0.27 | 0.58 | 0 |
| Waist circumference | Body size | 4.73 | 10.46 | 0 | 0.26 | 0.55 | 0 |
| Whistling and wheezing problems in the chest | OLD | 0 | 0 | 0 | 0 | 0 | 0 |
| Whistling and wheezing problems in the chest, last 12 months | OLD | 0.57 | 1.26 | 0 | 0 | 0 | 0 |
| Whistling and wheezing problems in the chest, only when having a cold, last 12 months | OLD | 0 | 0 | 0 | 0 | 0 | 0 |
| Whistling, wheezing at the same time as feeling short of breath, last 12 months | OLD | 0 | 0 | 0 | 0.13 | 0 | 0.25 |
| Widow | Living together with others | 0 | 0 | 0 | 0 | 0 | 0 |
| Work situation: emotionally demanding | Employment | 0 | 0 | 0 | 0 | 0 | 0 |
| Work situation: emotionally involving | Employment | 0.6 | 0 | 1.1 | 0.45 | 0.96 | 0 |
| Work situation: fast work pace | Employment | 0 | 0 | 0 | 0 | 0 | 0 |
| Work situation: influence over decisions | Employment | 5.57 | 12.31 | 0 | 0 | 0 | 0 |
| Work situation: influence over what you do | Employment | 0.43 | 0 | 0.79 | 0.45 | 0.96 | 0 |
| Work situation: influence over workload | Employment | 0.64 | 1.41 | 0 | 0 | 0 | 0 |
| Work situation: support from closest superior | Employment | 0 | 0 | 0 | 0 | 0 | 0 |
| Work situation: support from colleagues | Employment | 0 | 0 | 0 | 0.52 | 0 | 0.98 |
| Work situation: workload unevenly distributed | Employment | 0 | 0 | 0 | 0.89 | 0 | 1.68 |
| Year of immigration to Sweden | Immigration | 0 | 0 | 0 | 0.46 | 0.98 | 0 |
| Year of smoke stop (derived variable) | Smoking | 0 | 0 | 0 | 0 | 0 | 0 |
| Years of coughing | Coughing | 0 | 0 | 0 | 0 | 0 | 0 |
| Years of regular dwelling at indoor workplaces were people smoke | Smoking | 0 | 0 | 0 | 0 | 0 | 0 |
| Years of snus usage | Other nicotine products than cigarettes | 1.34 | 2.96 | 0 | 0 | 0 | 0 |
| Years with phlegm problems | Coughing | 0 | 0 | 0 | 0 | 0 | 0 |

The importance score corresponds to the SHapley Additive exPlanations (SHAP) absolute mean divided by the models’ mean absolute error, which is comparable to the effect size. The SHAP absolute mean corresponds to the average degree of change from the mean score of the Short Form 12 for physical HrQoL (men: 53.4; women: 51.4) by a predictor variable among all participants. Abbreviations: CABG = coronary artery bypass graft; COPD = Chronic obstructive pulmonary disease; FEV1 = forced expiratory volume in 1 second; FVC = Forced vital capacity; Hb = haemoglobin; HbA1c = haemoglobin A1c; HDL = high-density lipoprotein; hsCRP = High-sensitivity C-reactive protein; IBD = inflammatory bowel disease; LDL = low-density lipoprotein; MEF50 = maximal expiratory flow at 50 % of the forced vital capacity; MI = myocardial infarction; OLD = obstructive lung disease; PCI = Percutaneous Coronary Intervention; SLE = Systemic lupus erythematosus; VCmax = Maximum vital capacity.
