# Supplementary figures and images for: Factors important for health-related quality of life in men and women: The population based SCAPIS study
Source: PLoS One. 2023 Nov 3;18(11):e0294030. doi: 10.1371/journal.pone.0294030 (PMC10624288; doi:10.1371/journal.pone.0294030)

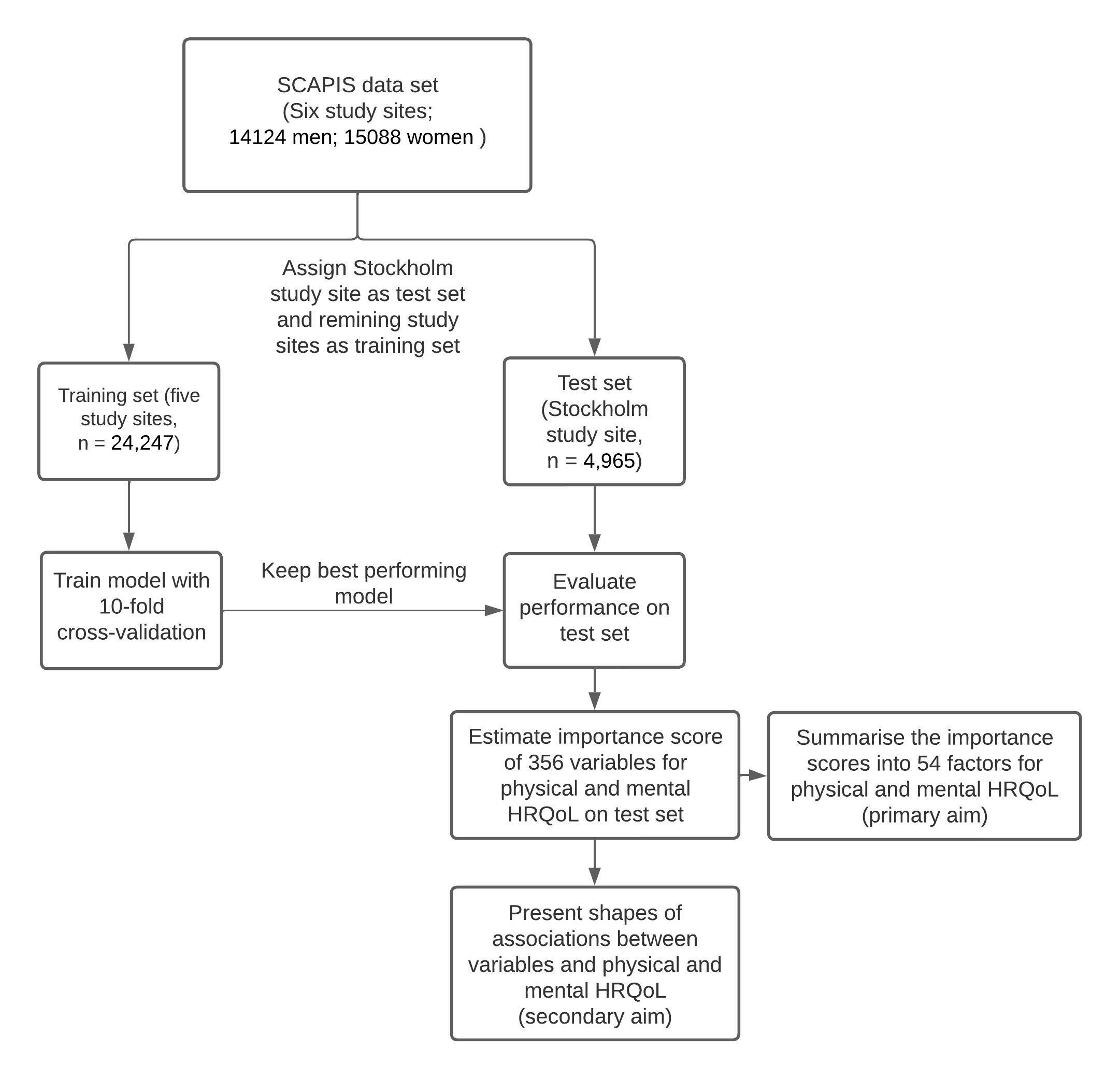

Supplement: S1 Fig — The Stockholm study site was used as the test sets. For the men´s physical and mental HRQoL models, the test sets comprised of 18% of the total men participant. For the women´s physical and mental HRQoL models the test sets comprised of 16% of the total women participants. (JPEG) [file pone.0294030.s005.jpeg]
